# Ca^2+^ efflux facilitated by co-transport of inorganic phosphate anion in the H^+^/Ca^2+^ antiporter YfkE

**DOI:** 10.1038/s42003-023-04944-6

**Published:** 2023-05-29

**Authors:** Wei Niu, Wenchang Zhou, Shuo Lu, Trung Vu, Vasanthi Jayaraman, José D. Faraldo-Gómez, Lei Zheng

**Affiliations:** 1 Department of Biochemistry and Molecular Biology, Center for Membrane Biology, the University of Texas Health Science Center at Houston McGovern Medical School, Houston, TX USA; 2 Theoretical Molecular Biophysics Laboratory, National Heart, Lung and Blood Institute, National Institutes of Health, Bethesda, MD USA

**Keywords:** Calcium, Molecular modelling, Transporters, Permeation and transport, Ion transport

## Abstract

Ca^2+^ is an important signaling messenger. In microorganisms, fungi, and plants, H^+^/Ca^2+^ antiporters (CAX) are known to play key roles in the homeostasis of intracellular Ca^2+^ by catalyzing its efflux across the cell membrane. Here, we reveal that the bacterial CAX homolog YfkE transports Ca^2+^ in two distinct modes: a low-flux H^+^/Ca^2+^ exchange mode and a high-flux mode in which Ca^2+^ and phosphate ions are co-transported (1:1) in exchange for H^+^. Coupling with phosphate greatly accelerates the Ca^2+^ efflux activity of YfkE. Our studies reveal that Ca^2+^ and phosphate bind to adjacent sites in a central translocation pathway and lead to mechanistic insights that explain how this CAX alters its conserved alpha-repeat motifs to adopt phosphate as a specific “transport chaperon” for Ca^2+^ translocation. This finding uncovers a co-transport mechanism within the CAX family that indicates this class of proteins contributes to the cellular homeostasis of both Ca^2+^ and phosphate.

## Abstract (web summary)

Bacterial CAX antiporter homolog YfkE can transport Ca^2+^ and inorganic phosphate (P_i_) together in a 1:1 ratio, exchanging them for H^+^ and becoming a high-flux antiporter when coupled with P_i_.

## Introduction

Calcium ions are among the most abundant metal ions in nature and are crucial for many important functions in cells, where Ca^2+^ serves as a versatile messenger^1,2^. Ca^2+^ signals are typically initiated by Ca^2+^ influx into the cytoplasm via selective membrane channels and is further augmented by Ca^2+^ efflux from intracellular organelles such as the endoplasmic reticulum. Sustained high levels of cytosolic Ca^2+^ are however lethal. Calcium-cation antiporters (CaCA) are key to restore Ca^2+^ homeostasis. This family of membrane transporters, which includes H^+^/Ca^2+^ antiporters (CAX) and Na^+^/Ca^2+^ exchangers (NCX), sequester and actively export Ca^2+^ out of the cytosol, powered by the influx of H^+^ or Na^+^ down transmembrane electrochemical gradients^3,4^.

CAXs are ubiquitous in bacteria, fungi, and plants^3^. For example, three CAX homologs, known as CAX1-3, have been identified in *Arabidopsis*, and more than twenty others in apple trees^3,5^. In plant cells CAX proteins are present in both the plasma and tonoplast membranes and facilitate the movement of cytosolic Ca^2+^ either out of the cell or back into the acidic vacuoles, in response to various stimuli including cold, salinity, and soil pH changes^5–8^. CAX homologs are also ubiquitous in microorganisms, although their physiological roles require further investigation. Ca^2+^ is involved in a broad range of bacterial processes including chemotaxis, cell division, and sporulation;^9^ Ca^2+^ signaling has also been implicated in bacterial infection and host–pathogen interactions^10^. CAX proteins are likely to play a central role in these processes, as they appear to be the major Ca^2+^ efflux systems in bacteria. Therefore, elucidating the transport mechanism of CAX will help understand how these organisms maintain Ca^2+^ balance in response to Ca^2+^ perturbations and signaling.

Structurally, all CAX proteins appear to share a common basic architecture, consisting of eleven transmembrane (TM) helices. Two conserved sequence motifs, referred to as α-repeats, are found in helices TM2-3 and TM7-8 and seem to provide sites for H^+^ and Ca^2+^ recognition. X-ray structures have recently begun to reveal the molecular basis for the mechanism of this class of antiporters, namely those of YfkE from *Bacillus subtilis*, VCX1 from *Saccharomyces cerevisiae*, and CAX_Af from *Archaeoglobus fulgidus*^11–13^. These structures suggest a mechanism wherein helices TM2-3 and TM7-8 adopt distinct arrangements that alternately expose recognition sites for H^+^ and Ca^2+^ to either side of the membrane. These three structures however capture a similar state in the alternating-access mechanism, namely one that is inwardly open (i.e., to the cytosol), in which two conserved glutamate residues important for the H^+^/Ca^2+^ exchange activity are adjacently located in a central ion-binding site. These two carboxylate residues coordinate one Ca^2+^ ion in the VCX1 structure^11^. Whether the structures of YfkE and CAX_Af capture an unliganded state or one bound to H^+^ was not immediately apparent, although the two carboxylate residues (E72 and E255 in YfkE) adopt different conformations compared to the Ca^2+^-bound state of VCX1. Recently, our studies for YfkE have also uncovered that its alternating-access mechanism is allosterically regulated by Ca^2+^ binding to an intracellular Ca^2+^ mini-sensor^12^.

Here, we reveal a type of Ca^2+^ transport mechanism for YfkE, and possibly other CAX family members. We demonstrate that YfkE features a transport mode wherein Ca^2+^ and inorganic phosphate (P_i_) are co-translocated in a 1:1 ratio, in exchange for H^+^. Coupling to P_i_ transforms YfkE from a low-flux to a high-flux antiporter. To gain insights into this Ca^2+^-P_i_ co-transport mechanism, we utilize multiple biochemical and biophysical approaches together with computer simulations and experiment-based molecular modeling. Our results lead us to conclude Ca^2+^ and P_i_ bind to a shared recognition site within the transporter interior, which includes E72 and E255 as well as other polar residues in the vicinity. E72 and E255 can however bind H^+^ instead, explain the ability of YfkE to harness the H^+^ gradient to drive Ca^2+^ and P_i_ transport. Our results also enable us to rationalize why translocation of P_i_ anions accelerates the Ca^2+^/H^+^ exchange reaction.

P_i_ is an important nutrient and is abundant in the cells for the synthesis of nucleotides, energy, and phospholipids. We provide an example that a transporter protein concurrently facilitates the membrane translocation of Ca^2+^ and P_i_. Our findings not only reveal a unique transport mode within the CAX family, but also suggest its physiological role in the homeostasis of both Ca^2+^ and phosphate in cells.

## Results

### YfkE co-transports Ca^2+^ and P_i_ in exchange for H^+^

**Fig. 1:**
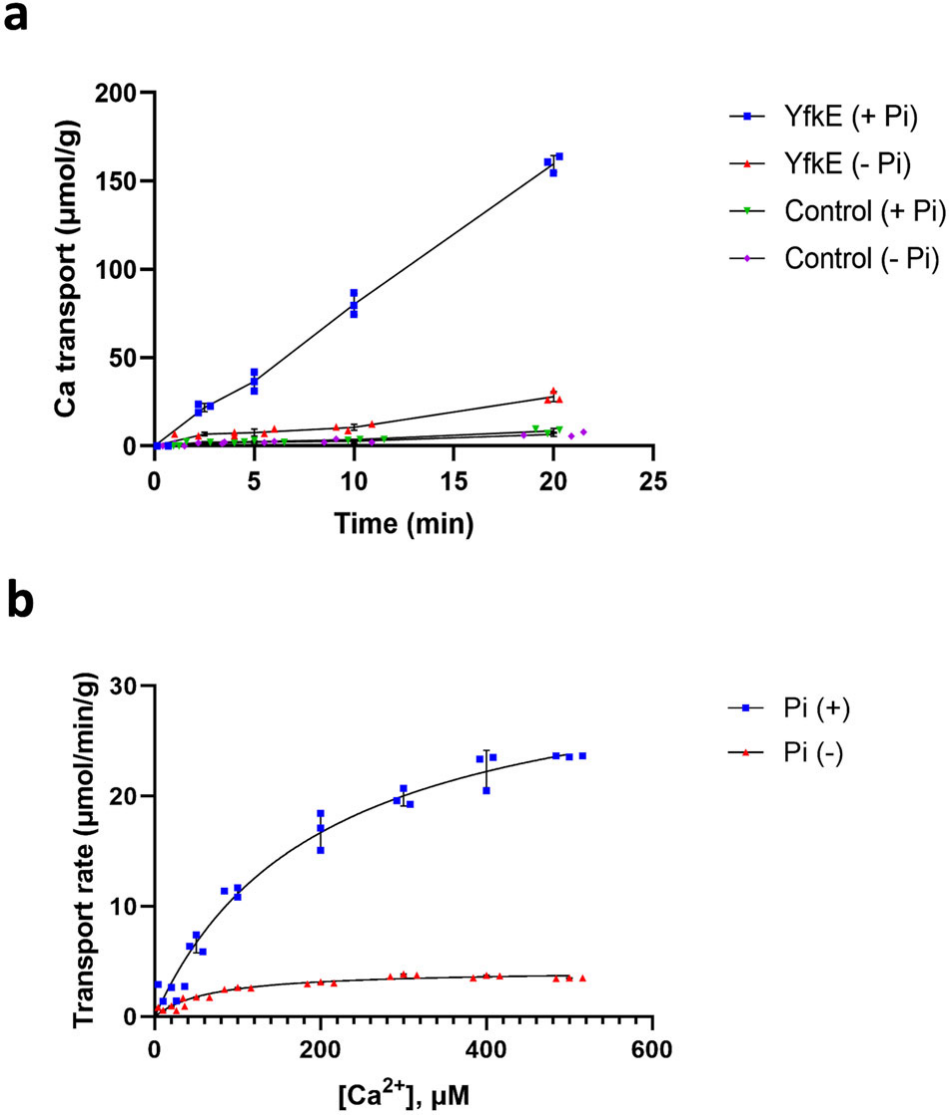
Ca^2+^ transport of YfkE facilitated by cytosolic inorganic phosphate ion (P_i_). **a** ^45^Ca^2+^ transport assays (+/− P_i_) measured using inside-out vesicles. Vesicles were mixed with 5 mM P_i_ before adding 0.5 mM ^45^CaCl_2_ to trigger the reactions at room temperature. Empty vesicles were used as control. **b** Kinetic analysis of Ca^2+^ transport of YfkE (+/−5 mM P_i_). Data were subtracted with the respective control vesicles and then fitted into the Michalis–Menten kinetics using GraphPad 9. Error bars represent standard deviations (*n*  =  3).

To begin to characterize YfkE, we measured its Ca^2+^ transport activity using inside-out vesicles, after an outward H^+^ gradient was established by adding NADH, which activates H^+^ pumping into the vesicles by the electron transport chain. As expected, the resulting time course showed that Ca^2+^ was transported into the YfkE vesicles (Fig. 1a). The observed transport activity fitted well to the Michaelis–Menten kinetics model, yielding *K*_M_ = 69 µM and *V*_max_ = 4.2 µmol/min/g (Fig. 1b). Strikingly, addition of 5 mM inorganic phosphate (P_i_) to the external solution (i.e., the intracellular side of the vesicle) strongly accelerated the Ca^2+^ transport activity of YfkE by eightfold (Fig. 1a). Specifically, kinetic analysis revealed *K*_M_ = 198.7 µM and *V*_max_ = 33.3 µmol/min/g (Fig. 1b). In contrast, we observed no effect for P_i_ analogs such as nitrate, sulfate, arsenate, and vanadate (Supplementary Fig. 1), or for ATP (Supplementary Fig. 2), indicating the acceleration of Ca^2+^ transport by YfkE is specifically mediated by P_i_. It is worth noting that Ca(H_2_PO_4_)_2,_ the major form of calcium phosphate compounds at neutral pH, is highly soluble in aqueous solutions. Consistently, we observed no Ca^2+^ accumulation in control vesicles devoid of YfkE even when P_i_ was present (Fig. 1a), ruling out that Ca^2+^ and P_i_ may form a precipitate. These results indicate that YfkE leverages the availability of cytosolic P_i_ to up-regulate its H^+^/Ca^2+^ antiport activity.

**Fig. 2:**
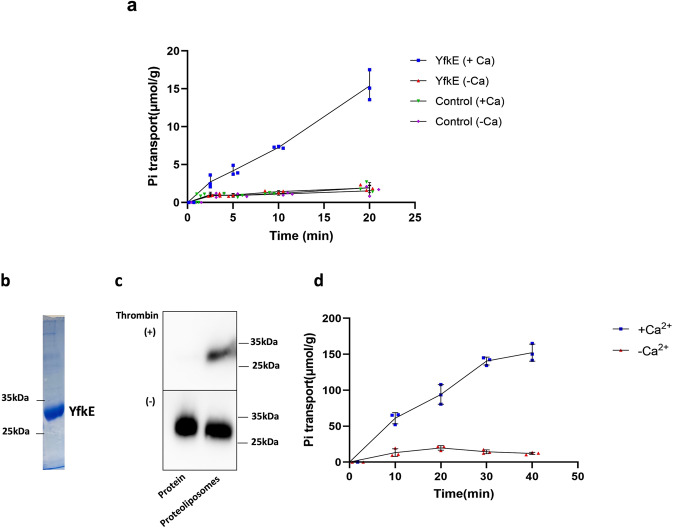
Co-transport of Ca^2+^ and inorganic phosphate ion (P_i_) by YfkE. **a** Time course of P_i_ transport of YfkE measured using inside-out vesicles (+/− 0.5 mM Ca^2+^). 5 mM ^32^P_i_ substrate was added to the reaction. Empty vesicles were used as control. **b** Coomassie-stained SDS-PAGE image of purified YfkE protein. **c** Assessment of the orientation of YfkE in proteoliposomes treated with thrombin. Immunoblot was developed using anti-His antibody. **d** ^32^P_i_ transport assays were measured using reconstituted proteoliposomes, showing that YfkE imports P_i_ in the presence of 0.5 mM Ca^2+^, not in the presence of 2 mM EDTA. Error bars represent standard deviations (*n*  =  3).

These observations led us to hypothesize that P_i_ stimulates the activity of YfkE because Ca^2+^ and P_i_ are co-transported. Although to our knowledge P_i_ transport has not been observed for other CAX antiporters, co-transport of a cation and P_i_ has been reported for other protein families. For example, the Na^+^/P_i_ symporter SLC34 uses a Na^+^ gradient to drive P_i_ uptake^14^. To test this hypothesis, we measured P_i_ transport using ^32^P-phosphate potassium as a substrate. As seen in Fig. 2a, P_i_ was imported into the YfkE vesicles in a time-dependent manner. Consistent with the Ca^2+^ transport assays (Fig. 1a), P_i_ uptake was only observed in the presence of Ca^2+^ (+0.5 mM) and no influx was detected when Ca^2+^ was absent (Fig. 2a). Similarly, no P_i_ uptake was detected in control vesicles lacking YfkE, whether in the presence or absence of Ca^2+^ (Fig. 2a). These results raised the hypothesis that YfkE catalyzes co-transport of Ca^2+^ and P_i_.

The vesicle assays described above demonstrate YfkE is required for co-transport of Ca^2+^ and P_i_, since this activity was not observed either in control vesicles lacking YfkE (Figs. 1a and 2a). To ascertain that YfkE alone mediates this activity without other ion-transport proteins being involved, we purified YfkE and reconstituted it into proteoliposomes (Fig. 2b, c and Supplementary Fig. 3). The orientation of YfkE in these proteoliposomes is likely to be right-side-out, assessed by limited proteolysis since thrombin would remove the N-terminal His-tag of YfkE on the intracellular surface (Fig. 2c and Supplementary Fig. 3). To initiate the transport reaction, an outward pH gradient was established by diluting the proteoliposomes (pH 7.4) into a higher pH buffer (pH 8), and H^+^-coupled P_i_ uptake was measured using P^32^-phosphate. The results clearly showed that YfkE imports P_i_ into the proteoliposomes in the presence of 0.5 mM Ca^2+^, but not in the absence of Ca^2+^ (+2 mM EDTA) (Fig. 2d). These results validate our vesicle assays and further confirm that YfkE alone co-transports Ca^2+^/P_i_ across the membrane in exchange for H^+^.

**Fig. 3:**
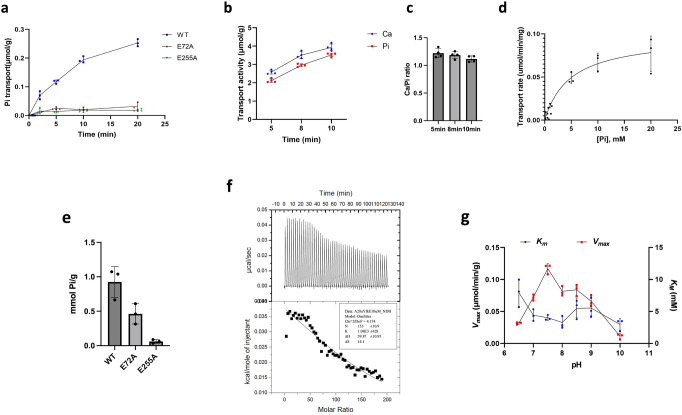
Characterization of phosphate (P_i_) binding to YfkE. **a** ^32^Pi transport assays were measured using inside-out vesicles, showing no activity of E72A (red squares) and E255A in contrast to WT. Assays were performed in the presence of 0.1 mM Ca^2+^. **b** Ca^2+^ or Pi transport rates measured in the same YfkE vesicles at indicated time points. **c** Stoichiometry of Ca^2+^ vs P_i_ by comparing their transport rates (**b**). **d** P_i_ transport kinetics of YfkE (+0.1 mM Ca^2+^) at indicated cytosolic pH conditions. Data were subtracted with (−) control and then fitted into the Michalis–Menten kinetics to calculate *K*_M_ and *V*_max_ using GraphPad 9. **e** Radiolabeled ^32^P_i_ binding assay using the purified YfkE protein wild-type, E72A, and E255A immobilized on Ni-NTA beads. **f** ITC analysis of P_i_ binding to YfkE. 10 mM P_i_ was titrated into a YfkE protein solution. The kinetic model fitting using Origin software yields a binding affinity of 0.93 ± 0.36 mM. **g** P_i_ transport kinetic parameters (*K*_M_ and *V*_max_) of YfkE measured at indicated pH. Error bars represent standard deviations (*n* = 3 for (**a**, **d**, **e**, **g**); *n* = 4 for (**b**, **c**)).

In accordance with the co-transport mechanism, the P_i_ transport activity of YfkE WT was significantly decreased when the Ca^2+^ concentration was reduced from 0.5 mM (Fig. 2a) to 0.1 mM Ca^2+^ (Fig. 3a). Comparison of the observed transport rates for each species in the same vesicles indicates that the stoichiometry of this coupling is 1 Ca^2+^:1 P_i_ (Fig. 3b, c). Compared to Ca^2+^, however, the apparent binding affinity of P_i_ is much weaker (*K*_M_ = 4.9 mM), based on analysis of the transport kinetics (Fig. 3d and Table 1). P_i_ substrate binding was also demonstrated using a radiolabeled ^32^P_i_ binding assay. The results showed P_i_ binding to the YfkE WT protein, but it was disrupted by the mutation of E72A and E255A (Fig. 3e). P_i_ binding was further characterized by isothermal titration calorimetry (ITC), yielding a binding affinity of ~1 mM (Fig. 3f) in line with the *K*_M_ value generated from the transport kinetic analysis (Fig. 3d). Together with the above transport assays, all these in vitro characterizations collectively confirm that P_i_ is indeed a co-transport substrate of YfkE.

**Table 1:** Phosphate transport kinetics of YfkE WT and mutants.

	WT	E72A	E255A	R3A	R36A	N99A	N252A	Q281A	H256A
*K*_M_ (mM)	4.9	N.D.	N.D.	5.5	4.9	12.4	8.2	29.9	34.2
*V*_max_ (μmol/min/g)	0.097	N.D.	N.D.	0.075	0.041	0.039*	0.019*	0.019*	0.051*

Note: the kinetic values were generated from the data in Figs. 3d and 6a and Supplementary Fig. 4b, c using Graph Prism 9. *These mutations disrupted transport activity. Therefore, different concentrations (5x higher than WT) of vesicles were used to obtain useful data for kinetic analysis. Only *K*_M_ values are compared in this table.

Taking together, these data lead us to conclude that YfkE catalyzes Ca^2+^ efflux using two different modes or mechanisms: a slow mode, when P_i_ is absent or largely unavailable; and a fast mode, when intracellular P_i_ is sufficiently abundant, which involves co-transport of Ca^2+^ and P_i_. Importantly, in both cases Ca^2+^ transport requires counter-transport of H^+^, down its electrochemical gradient, in accordance with the alternating-access model. Accordingly, addition of the H^+^ ionophore carbonyl cyanide m-chlorophenyl hydrazone (CCCP) to our transport assays completely abrogated Ca^2+^ uptake into the inside-out vesicles, in the absence or the presence of P_i_ (Supplementary Fig. 2).

In solution, P_i_ can exist in different states of ionization depending on pH. In particular, the equilibrium between mono-anionic H_2_PO_4_^1−^ and di-anionic HPO_4_^2−^ has a pK_a_ of 7.2. To evaluate what species of P_i_ is the most likely to be co-transported with Ca^2+^ by YfkE, we measured P_i_ uptake into inside-out vesicles varying the external pH, that is, varying the magnitude of the outward H^+^ gradient, but also the P_i_ ionization equilibria. As expected, P_i_ uptake accelerated as the external pH was increased from 6.5 to 7.5, likely reflecting a stronger driving force from downhill H^+^ efflux (Fig. 3g). A greater pH values, however, transport was clearly inhibited, in a gradual manner, indicating that YfkE cannot recognize di-anionic P_i_ but rather mono-anionic H_2_PO_4_^1−^.

### Ca^2+^ and P_i_ share a central recognition site in YfkE

Unlike Ca^2+^, there is no clear definition of a prototypical P_i_ binding motif in protein structures. However, it is known that P_i_ binding is favorably contributed by positively charged residues (arginine and lysine) and polar residues such as threonine, serine and histidine^15^. The Ca^2+^ recognition site in the interior of YfkE, formed by E72, E255 and surrounding polar residues, is the only region suitable for P_i_ recognition in the transmembrane domain of the protein (Supplementary Fig. 4a)^12^. Attempts to identify an alternative pathway for P_i_ translocation, for example, mediated by R4 and R46, two positively charged residues on the intracellular protein surface, resulted in no alteration of the P_i_ transport activity (Supplementary Fig. 4b, c and Table 1). As mentioned above, radiolabeled P_i_ binding assays show that mutation of the two residues known to coordinate Ca^2+^ also disrupted P_i_ binding (Fig. 3e), indicating these two ions bind in proximity to each other, in a shared recognition site within the protein.

**Fig. 4:**
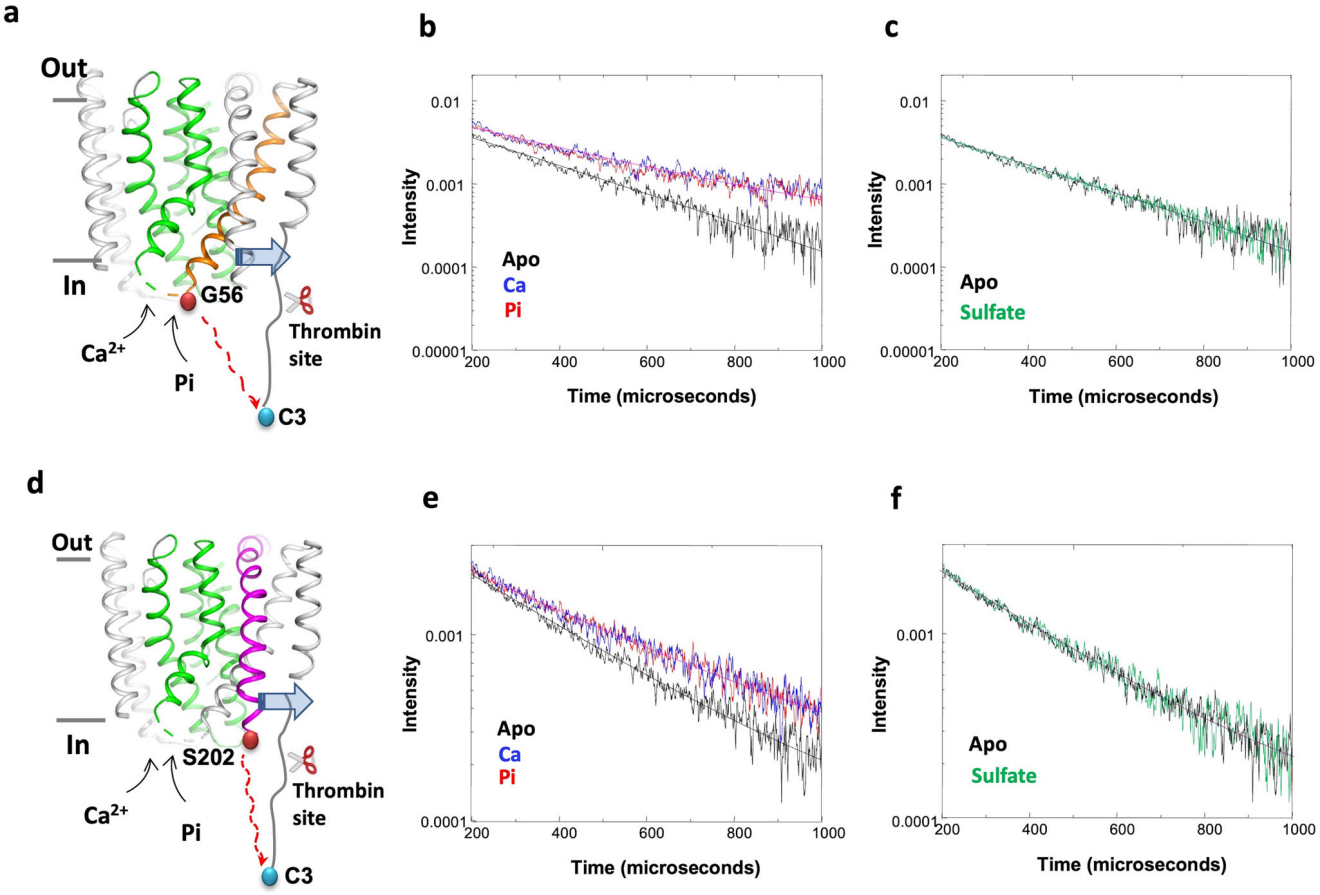
Conformational changes of YfkE induced by Ca^2+^ and inorganic phosphate ion (P_i_). **a**, **d** Theme of LRET experimental design to measure the distance (red dashed line) between the donor (blue sphere) at N-terminal C3 and receptor (red sphere) at G56C from TM1 (orange) (**a**) or S202C from TM6 (magenta) (**d**). Pathway-forming helices are colored in green; Thrombin cleavage (shown as a scissor) removes the donor to calculate the background of the raw membrane. Blue arrows indicate helix conformational changes in the membrane. **b**, **c**, **e**, **f** LRET traces after subtracting respective background: apo form (black), +0.5 mM Ca^2+^ (blue), +5 mM P_i_ (red), and 5 mM sulfate (green). **b**, **c** G56C; **e**, **f** S202C.

To further test this hypothesis, we developed an assay based on luminescence resonance energy transfer (LRET). LRET measurements report on changes in the distance between a fluorescent donor and its acceptor, manifested as luminescence signals of different lifetimes. Through this approach, we sought to ascertain whether recognition of Ca^2+^ and P_i_ induce comparable conformational changes on YfkE. Like the functional assays described above, these measurements were carried out in inside-out vesicles, to examine the protein in its native membrane environment (see 'Methods' for details). Specifically, we labeled positions G56 (intracellular TM1-2 loop) and C3 (amino-terminus) with terbium chelate and Atto-465, respectively, using thiol-reactive chemistry (Fig. 4a). Based on the LRET results, the distance between donor and acceptor in the absence of any substrate is 25.5 ± 0.2 Å (Fig. 4b). Addition of either 0.5 mM Ca^2+^ or 5 mM P_i_ to the vesicles increased the distance similarly, to 26.9 ± 0.3 Å. To verify this result, we introduced the terbium chelate donor at position of S202 (in TM6), while keeping the Atto-465 acceptor in C3 (Fig. 4d). As observed for G56, adding Ca^2+^ or P_i_ increased the distance between donor and acceptor similarly, from 26.3 ± 0.1 Å to 27.7 ± 0.2 or 28 ± 0.2 Å (Fig. 4e). Importantly, both G56C and S202C mutants retained Ca^2+^ uptake activity in the presence of P_i_ (Supplementary Fig. 5). Therefore, the distance changes detected by LRET likely reflect protein conformational changes induced by substrate binding. Admittedly these changes are modest, due to a suboptimal position of donor and acceptor, and thus it is not possible to discern what protein motions are represented by this data. However, these differences are statistically significant and specifically induced by Ca^2+^ or P_i_. Indeed, no changes were observed upon addition of 5 mM sulfate, a P_i_ analog (Fig. 4c, f). In conclusion, the LRET measurements support our hypothesis that both Ca^2+^ and P_i_ access a common recognition site within the protein.

### Inward-facing structure of YfkE captures H^+^ bound state

To provide a structural context to these biochemical and biophysical measurements, we resorted to molecular modeling. Specifically, we sought to construct a hypothetical model that might explain how YfkE recognizes both Ca^2+^ and P_i_ in a manner that results in competition with H^+^, as is the norm for a coupled ion antiporter. The first step in this process was to determine whether the existing structure of inward-facing YfkE^12^ captures a H^+^-bound state. If so, we reasoned that this experimental structure would provide a good foundation to model the state bound to Ca^2+^ and P_i_; indeed, available structural data for membrane antiporters show that alternate substrate-bound forms of the same functional state differ primarily in the configuration of the sidechains forming the substrate binding sites, with no major changes in the protein backbone.

**Fig. 5:**
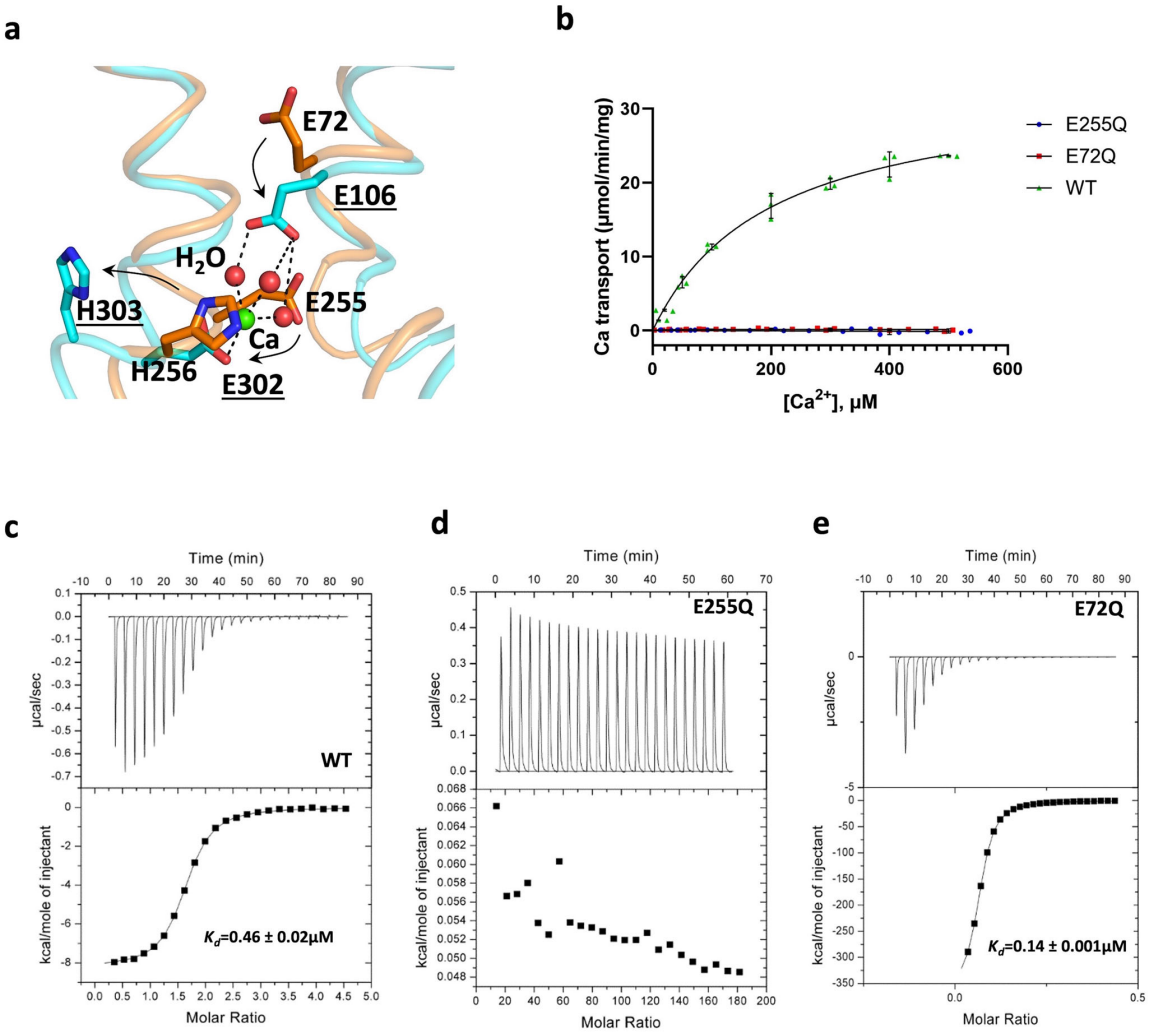
Distinct roles of E72 and E255 in the H^+^/Ca^2+^ exchange mechanism of YfkE. **a** Comparison between the structures of protonated YfkE (cyan) and Ca^2+^-bound VCX1 (orange), showing their conformational changes (black arrows) of three titratable residues (sticks) in the translocation pathway. TMs 2 and 7 are depicted as cartoons. In VCX1, E106 interacts with Ca^2+^ (green sphere) via waters (red sphere) by H-bonds (black dashed lines). The residues of VCX1 are labeled underlined. **b** ^45^Ca^2+^ transport kinetic analysis measured using inside-out vesicles showing no activity of E72Q and E255Q in contrast to WT. Data were subtracted with (−) control vesicles. Error bars represent standard deviation (*n* = 3). **c**–**e** Isothermal titration curves of Ca^2+^ into a protein solution of YfkE WT (**c**), E255Q (**d**), and E72Q (**e**). Data fitting was carried out using the software Origin.

To that end, we carried out a series of all-atom molecular dynamics simulations designed to quantify the likelihood of protonation of selected side chains within the transporter interior (Supplementary Fig. 6). Specifically, we focused on E72, E255, and H256, the three protonatable residues in the central pathway within the α-repeats (Fig. 5a). H256 had been previously predicted to be involved in H^+^ binding since it is conserved in H^+^-coupled CAXs, but not in Na^+^-coupled NCXs^12^. As negative controls, we also probed E233 and H226, which are exposed on the protein surface and are thus not expected to deviate significantly from their inherent protonation propensities in solution, for a given pH. As summarized in Table 2, our results strongly indicate that both E72 and E255 are concurrently protonated in the existing inward-facing structure of YfkE. When protonated and deprotonated states are compared individually, it is most clear that the former is very strongly favored in the specific geometry captured in the crystal structure, relative to what is intrinsic for this type of sidechain in solution. (In other words, the ‘apparent pKa’ of both E72 and E255 in this specific conformation is strongly shifted upwards.) This propensity is considerably greater for E72 than E255, suggesting that E72 is the last site to deprotonate in the inward-facing state. If E255 is re-evaluated while E72 is protonated (i.e., uncharged), the protonation probability of E255 is logically diminished, but it remains strongly up-shifted. By contrast, the calculated protonation propensity for the surface residue E233 is very similar to that of free glutamate in solution, whether or not E72 and E255 are assumed to be protonated. This result is what is expected for a non-functional site, corroborating the validity of the simulation methodology used here to evaluate relative protonation energetics.

**Table 2:** Evaluation of the protonation state of the crystal structure of YfkE.

	E72	E255	H256	E233	H226
Single protonation^a^	–11.5 ± 0.5	–7.1 ± 0.4	0.3 ± 0.1	–0.5 ± 0.1	0.9 ± 0.1
With E72 protonated^b^		–5.1 ± 0.6	1.8 ± 0.7		
With E72, E255 protonated^c^			3.3 ± 1.2	–0.2 ± 0.1	0.3 ± 0.3

Note: The table reports the increase or decrease in the free-energy of protonation (ΔΔ*G*) of selected sidechains in YfkE, relative to the corresponding sidechain in solution, calculated with all-atom MD simulations using the Free-Energy Perturbation method ('Methods' and Supplementary Fig. 6). All values are in kcal/mol. Values are shown for calculations wherein (a) one sidechain is evaluated while all others are assumed to be deprotonated; (b) E72 is set in the protonated state and other sidechains are evaluated; and (c) both E72 and E255 are set in the protonated state. For reference, in this context a ΔΔ*G* value approximately equal to –1.4 kcal/mol corresponds to a pK_a_ shift of +1 pH unit.

In contrast to our findings for E72 and E255, examination of H256 using the same approach indicates this sidechain is deprotonated in the conformation of YfkE captured by the crystal structure (Table 2). Even when E72 and E255 are assumed to be negatively charged, which would in principle favor a positive charge at H256, we detect no significant shift in the protonation probability of H256 relative to a histidine analog in solution, or relative to H226, which is exposed on the protein surface. When E72 and E255 are assumed to be protonated, protonation of H256 is even less likely; indeed, deprotonated H256 is clearly favored (i.e., its ‘apparent pKa’ is down-shifted).

In conclusion, this analysis indicates that the inward-facing crystal structure of YfkE captures a substrate-bound state, namely with two H^+^ bound at sites E72 and E255. H256, however, does not concurrently bind a third H^+^, suggesting the antiport stoichiometry of YfkE might be 1Ca^2+^:2H^+^. These results are consistent with the abovementioned observation that alanine mutation of either E72 or E255 abrogates H^+^-driven Ca^2+^ transport in experimental assays^12^. By contrast, the effect of alanine mutation of H256, while significant, is comparable to that of mutation of other non-protonatable polar side chains in the vicinity of H^+^ binding sites, such as N69A or Q281A^12^.

### Distinct roles of E72 and E255 in competitive binding of H^+^and Ca^2+^

As mentioned, structural studies of a CAX protein from yeast, VCX1, reportedly detected a Ca^2+^ ion bound^11^. The binding site is formed by two acidic residues, E109 and E305, which are equivalent to E72 and E255 in YfkE, respectively. It seems clear, therefore, that 2 H^+^ compete with 1 Ca^2+^ for binding to this site. However, the different protonation propensities of E72 and E255 suggest they are not alike in this competition mechanism. In fact, the structure of VCX1 also indicates the different roles of these two carboxylate residues in Ca^2+^ binding, i.e., E305 (E255 in YfkE) directly coordinates Ca^2+^, while E109 (E72 in YfkE) indirectly interacts with the ion via three water molecules (Fig. 5a)^11^. To gain insights into their specific roles in Ca^2+^/H^+^ binding, we mutated E72 and E255 to glutamine, by which deprotonation is eliminated while Ca^2+^ binding remains possible in principle. As expected, either the E72Q or E255Q mutation completely abolished Ca^2+^ transport, as deprotonation of both carboxylate side chains is required to close the antiport cycle (Fig. 5b). However, examination of these two mutant proteins using ITC confirmed they have distinct roles in terms of H^+^/Ca^2+^ binding. Ca^2+^ titration to WT YfkE yielded a binding affinity of 0.46 ± 0.02 µM (Fig. 5c). No saturation was observed for E255Q within the titration range, indicating the E255 carboxylate group is required for Ca^2+^ binding (Fig. 5d). In sharp contrast, the carboxylate in E72 appears to be dispensable since E72Q does not impair Ca^2+^ binding. Instead, the mutation increases the apparent Ca^2+^ binding affinity threefold (*K*_d_ = 0.14 ± 0.001 µM) compared to WT (Fig. 5e). Taken together, computational and experimental results suggest that the first step in the mechanism by which YfkE recognizes Ca^2+^ in the inward-facing state is the deprotonation of E255; this step is necessary and sufficient for Ca^2+^ binding. Deprotonation of E72 would occur subsequently and likely be followed by a larger-scale conformational transition to the outward-facing state.

### Hypothetical structural basis for the concurrent recognition of Ca^2+^ and P_i_

**Fig. 6:**
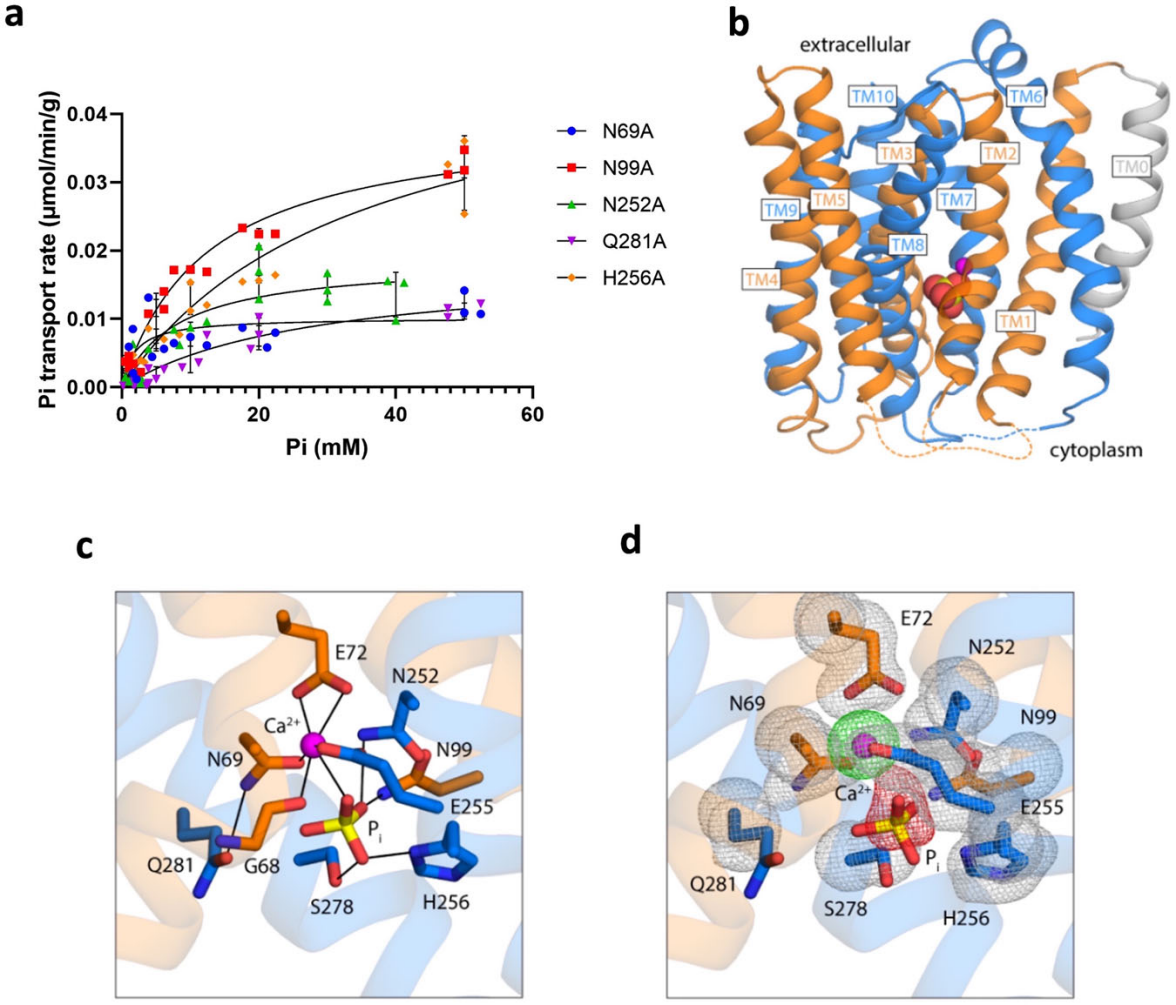
Proposed mode of recognition Ca^2+^ and inorganic phosphate ion (P_i_) by inward-facing YfkE. **a** P_i_ transport kinetic assays measured using inside-out vesicles: N69A, N99A, N252A, Q281A, and H256A. Different concentrations (5× higher than WT) of vesicles were used for mutants to obtain useful data for kinetic analysis. Data were subtracted with (−) control vesicles and then fitted into the Michalis–Menten kinetics using GraphPad 9. Error bars represent standard deviations (*n* = 3). **b** Overview of the YfkE model viewed along the membrane plane; the two inverted-topology repeats, each comprising five transmembrane helices, are colored in orange (TM1-TM5) and marine blue (TM6-10). A transmembrane helix (TM0) precedes the N-terminal repeat. The binding sites for Ca^2+^ (magenta sphere) and P_i_ (yellow and red spheres) are formed by residues in the so-called alpha-repeats, i.e., TM2-TM3 and TM7-TM8. **c** Close-up of the hypothetical structure of the binding site for Ca^2+^ and P_i_ in YfkE. Residues involved in ion coordination are highlighted. The proposed interaction network is indicated with black lines. **d** Same as **c**, with overlaid occupancy maps calculated from the ensemble of models generated in this study. Specifically, the figure shows a contour of the occupancy maps at a 85% value, for both the protein side-chains (gray mesh), Ca^2+^ (green mesh), and P_i_ (red mesh). That is, the portion of the structure inside the contours is roughly consistent across 85% of the ensemble, while the portion outside is variable.

Multiple lines of evidence support that both Ca^2+^ and P_i_ are adjacently bound in the central translocation pathway: first, mutation of the residues involved in Ca^2+^ binding, i.e. E72 and E255, not only abrogate P_i_ transport (Fig. 3a) but also P_i_ binding (Fig. 3e); second, Ca^2+^ and P_i_ induce similar conformational changes as measured by LRET (Fig. 4); and third, P_i_ co-transport alters the *K*_M_ for Ca^2+^ (Fig. 1b). In the structure of YfkE, several polar residues including N69, N99, Q252, Q281, and H256 are located adjacent to E72 and E255 in the vicinity of the transport binding site^12^. Although these polar residues are conserved in the CAX family (Supplementary Fig. 7), they have no direct interaction with Ca^2+^ in the Ca^2+^-bound state structure of VCX1^11^. We hypothesize these residues are involved instead in P_i_ binding. To test this hypothesis, we examined the effects of alanine mutation of those polar residues using P_i_ transport assays (Fig. 6a). The role of each residue in P_i_ binding was assessed by transport *K*_M_ analysis (Table 1). The most significant changes in *K*_M_ were found for Q281A and H256A, showing a reduction by six- or sevenfold compared to WT, indicating an important role in P_i_ recognition. It should be noted that these mutations also disrupt Ca^2+^ uptake^12^, in line with our hypothesis that the binding sites for Ca^2+^ and P_i_ are proximal.

Having established that the existing structure inward-facing YfkE represents a H^+^-bound state, rather than an apo conformation, we proceeded to model the changes in this structure that might explain how Ca^2+^ and mono-anionic P_i_ are concurrently recognized and transported, after H^+^ are unloaded. To that end, we used a procedure akin to conventional homology modeling, adapted to only consider solutions that satisfy a series of geometric restraints reflecting the biochemical and functional data we have obtained for this antiporter (see 'Methods' for further details). The model also incorporates information derived from a survey of the Protein Data Bank, specifically from a protein of known structure that also recognizes Ca^2+^ and P_i_ concurrently, namely a Ca^2+^ -dependent hydrolase known as PON1^16^. This protein exhibits a donut-shaped structure with a central site where Ca^2+^ and P_i_ are coordinated, which appears strikingly similar to the binding site in YfkE and VCX1 (Supplementary Fig. 8).

To adequately explore the range of side-chain configurations that are compatible with the sets of restraints enumerated above, we generated an ensemble of 2000 models in total. We then analyzed this conformational ensemble using a clustering algorithm based on pairwise similarity (see 'Methods' for further details). The model shown in Fig. 6b–d is representative of the most populated cluster, which includes ~52% of all models produced. By design, the model shows minimal deviations in the conformation of the backbone relative to the experimental structure of the H^+^-bound state. Nonetheless, it seems clear that a rearrangement of a limited number of sidechains is sufficient to produce a model that is not only plausible chemically but also consistent with existing experimental data. Specifically, Ca^2+^ is coordinated through concurrent bi-dentate interactions with the carboxylate groups of E72 and E255 as well as by the carbonyls of G68 and N69. P_i_ is in close proximity and is coordinated by multiple residues acting as hydrogen-donors, in addition to H256, which seems poised to act as an acceptor. While this inward-facing model is by definition hypothetical, we posit it provides a plausible structural context to our biochemical and physiological data and shows that concurrent recognition of Ca^+^ and P_i_ at a shared site within YfkE is entirely feasible.

## Discussion

Fluctuations in cytosolic Ca^2+^ concentration are a universal cellular signaling strategy. Ca^2+^-mediated signaling is however not only dependent on the magnitude of the changes in concentration but also on the specific rate, frequency, and spatiotemporal patterns of those changes. Generation of these complex signals in response to stimuli requires the concerted action of Ca^2+^ channels, transporters, and pumps on the plasma membrane and the membranes of intracellular organelles, so as to regulate Ca^2+^ influx and efflux^9,17^. In unicellular organisms and higher plants, CAX antiporters provide one of the primary mechanisms for maintaining this complex Ca^2+^ balance^3,18^.

In this study, we reveal a type of the regulatory mechanism of CAX. We found that the H^+^/Ca^2+^ exchange activity of the bacterial CAX homolog YfkE can be greatly accelerated in the presence of P_i_. Furthermore, our results using both *E. coli* vesicles (Fig. 2a) and reconstituted proteolipsomes (Fig. 2d) strongly indicate that YfkE co-transports P_i_ with Ca^2+^; that is, P_i_ might be thought as a “transport chaperon” that facilitates Ca^2+^ efflux. Based on our transport assays, the coupling of Ca^2+^ and P_i_ is highly specific (Supplementary Fig. 1), but these two ions exhibit distinct affinity, i.e., the apparent *K*_M_ for Ca^2+^ is 0.15 mM (Fig. 1b), while that for P_i_ is 5 mM (Fig. 3c), which is also demonstrated by the ITC experiments (Fig. 2f). This disparity may be a reflection of their different concentrations in cells. Cytosolic [Ca^2+^] is tightly controlled below μM^9,19^, whereas P_i_ is much more abundant, in both prokaryotic and eukaryotic cells, at 1–10 mM^20,21^. The abundancy of cytosolic P_i_ should enable YfkE to co-transport Ca^2+^ and P_i_ in typical physiological conditions.

P_i_ is not only an important component in cellular metabolisms but also serves as a signaling molecule^22^. Multiple P_i_ transport systems are available to facilitate phosphate homeostasis. In bacteria and yeast, specific P_i_-binding proteins respond to changes in ambient P_i_ availability at the plasma membrane and transduce intracellular signals to up-regulate the expression of genes involved in P_i_ uptake^23,24^. Our finding that the Ca^2+^ efflux activity of YfkE is regulated by the availability of cytosolic P_i_ raises the hypothesis that CAX proteins are involved in the homeostasis of both Ca^2+^ and P_i_, two essential ions in the cells. It was previously suggested that YfkE is involved in sporulation in *Bacillus subtilis*^25^. Interestingly, Ca^2+^ accumulation was found as a calcium phosphate salt in retrogressive *Bacillus* spores^26^. It is possible that YfkE utilizes its Ca/P_i_ co-transport activity to regulate this process. All these hypotheses require further investigation, but it is worth noting that Ca^2+^-P_i_ co-transport has been described in both bacteria and plants. Rosen et al. found that Ca^2+^ efflux in *E. coli* is mediated by two transport systems, one P_i_ independent and another P_i_ dependent^27^. In *Arabidopsis*, disruption of the vacuolar H^+^/Ca^2+^ transporters CAX1 and CAX3 causes notable alterations in P_i_ content^28^. Our biochemical, biophysical and computational analyses indicate that YfkE recognizes Ca^2+^ and P_i_ in a common site, through direct or indirect interactions with residues that are conserved among many CAX proteins, including E72, E255, N69, N99, Q252, Q281, and H256 (Supplementary Fig. 7). Therefore, it seems plausible that the co-transport mechanism we observe for YfkE may be a common feature within the CAX family.

**Fig. 7:**
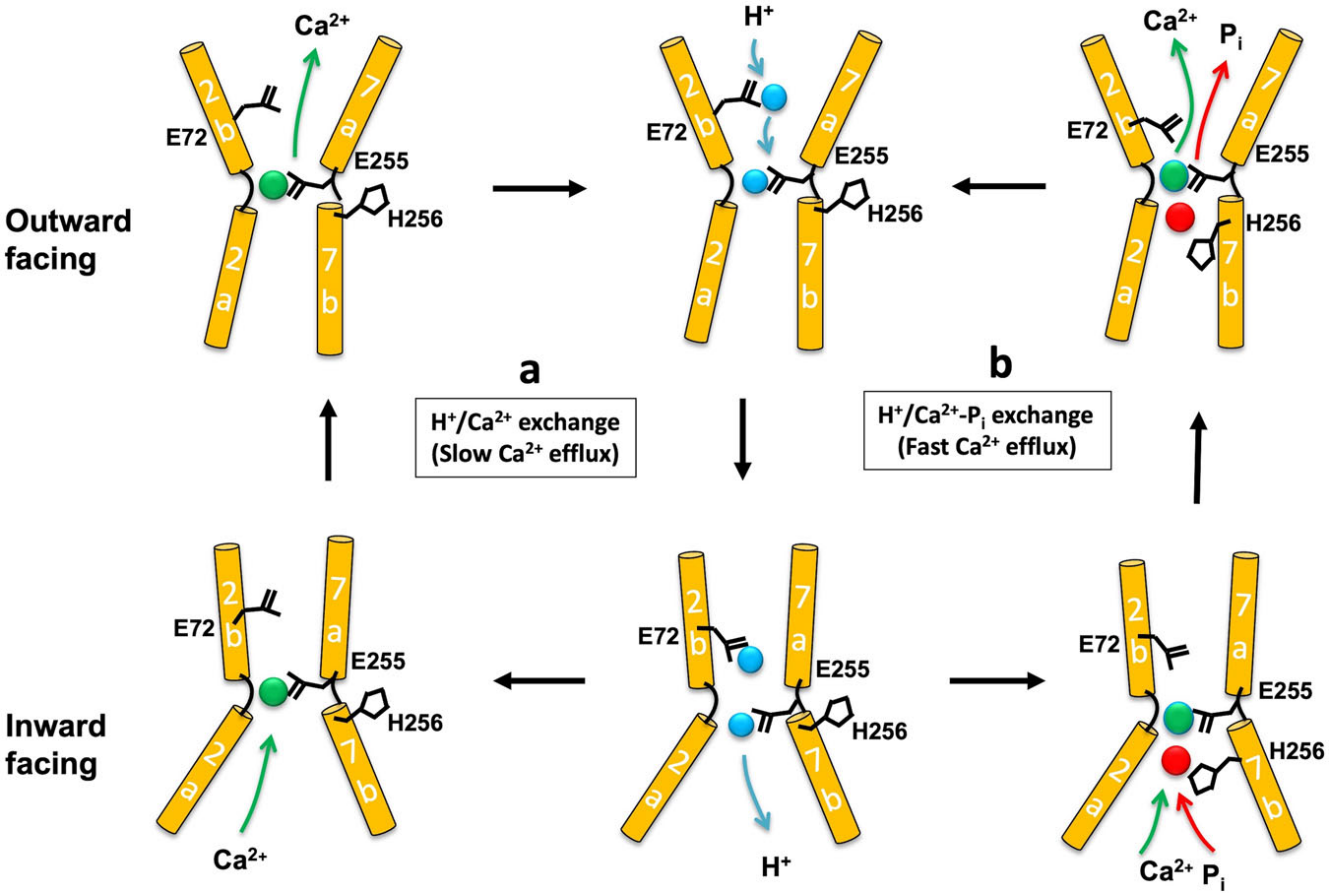
Thematic models of the two Ca^2+^ transport modes of YfkE. **a** YfkE maintains a low-efflux transport mode in the absence of P_i_, in which the alternating access of H^+^/Ca^2+^ triggers the conformational transition between the inward-facing and outward states. **b** The presence (or increase) of cytosolic P_i_ promotes YfkE to a high-flux H^+^/Ca-P_i_ co-transport mode. In both modes, E72 transfers H^+^ (green sphere) to E255 to dissociate Ca^2+^ (blue sphere). In the co-transport mode, P_i_ (red sphere) bound between E255 and H256 adjacent to Ca^2+^ facilitates transport turnover.

While the transport mechanism of YfkE and other CAX proteins is likely to be accurately described by the alternating-access model, a confirmation will require further structural information beyond the existing inward-facing states. Our functional and structural analyses reveal a regulatory mechanism of YfkE activated by the availability of cytosolic P_i_. In the absence or poor abundance of P_i_, YfkE functions in a low-efflux H^+^/Ca^2+^ exchange mode involving ion competition for E72 and E255, the two carboxylate residues in the central site (Fig. 7a). Both E72 and E255 are functionally essential. As noted, our computational results show that YfkE carries the transported H^+^ at E255 and E72 (Table 2). Our ITC experiments (Fig. 5c–e) suggest that initial Ca^2+^ binding to the inward-facing state requires displacement of the H^+^ bound at E255 but not necessarily at E72 (Table 2). Both protons must be however released before the transporter can return to the outward-facing state. It is possible therefore that the complete deprotonation of the central site following Ca^2+^ binding to a partially protonated form is a limiting factor from a kinetic standpoint.

Elevation of cytosolic [P_i_] switches the transporter to a high-flux mode by coupling Ca^2+^ efflux to the co-transport of P_i_, with both ions adjacently recognized in the central site in the pathway (Fig. 7b) as suggested by our docking model and mutational studies (Fig. 6). The mechanism by which P_i_ accelerates Ca^2+^ efflux requires further investigation. When cytosolic P_i_ is sufficiently abundant, P_i_ might alter the thermodynamics of the H^+^/Ca^2+^ exchange reaction, which would be energized by the outward transmembrane gradient of P_i_ in addition to the inward H^+^ gradient. Alternatively, or in addition, P_i_ binding in addition to Ca^2+^ might help accelerate the complete unloading of H^+^ from the transporter, thereby enabling it to transition between inward and outward-facing states while bound to Ca^2+^. This hypothesis is supported by the kinetic analyses in which the coupling of P_i_ leads to a large increase of *V*_max_ of transported Ca^2+^ by 8x while the binding affinity of transported Ca^2+^ (*K*_M_) is modestly reduced. Bound P_i_ does not interact with E72 directly, according to our hypothetic structural model, but it is proximal to E255 (Fig. 6c, d). Thus, it is possible that the release of H^+^ from E72 occurs via E255, and that mono-anionic P_i_ serves a transient protonation site between E255 and H256, which would then unload the H^+^ into the cytosol. P_i_ would therefore accelerate transport turnover by expediting the complete H^+^ dissociation from the inward-facing state. Of note, the *V*_max_ of P_i_ transport peaks at pH 7.5, which is very close to the pK_a_ (7.2) of the equilibrium between mono-anionic H_2_PO_4_^1−^ and di-anionic HPO_4_^2−^ species (Fig. 3g). Interestingly, a P_i_-mediated H^+^ transfer mechanism (between two carboxylate residues) has also been proposed for a H^+^/phosphate transporter^29^. It is thus conceivable that this kind of H^+^ hopping mechanisms are common among P_i_-coupled transporters. Despite the availability of multiple structures of CAX^11–13^, the stoichiometry of H^+^ vs Ca^2+^ has been elusive since its determination remains technically challenging. In this study, we found that the transport activity of a CAX homolog is regulated by the presence of P_i_, which is itself a protonatable species. Careful characterization of H^+^ coupling in each transport mode will therefore be required to dissect the interplay between P_i_, Ca^2+^ and H^+^ in this exchange mechanism.

YfkE belongs to the CaCA superfamily, which thus far includes more than 200 members in all domains of life^18^. All CaCA homologs feature the two sequence motifs known as α-repeats (TM2-3 and TM7-8) featuring the two carboxylate residues used for H^+^/Na^+^/Ca^2+^ coordination (e.g., E72 and E255 in YfkE). These motifs are however not strictly conserved, and sequence variations therein appear to modulate the substrate selectivity of different transporters and ultimately dictate whether the coupling ion in the exchange cycle is H^+^ or Na^+^^11–13,30^ (Supplementary Fig. 9). In YfkE, protonation of both E72 and E255 in the outward-facing state enables the transporter to reach the inward-facing conformation, which can then load cytosolic Ca^2+^ (and P_i_). E54 and E213, the two counterpart carboxylate residues in the Na^+^/Ca^2+^ exchanger NCX_Mj, can also bind H^+^ in the outward-facing state, but the transporter is then inhibited^31,32^ as it cannot access the inward-facing state^33^, in line with the fact that NCX_Mj does not transport H^+^^30,33^. In contrast, Na^+^ binding facilitates that conformational transition enabling NCX_Mj to harness the inward Na^+^ gradient. The correspondence between transport and binding specifies is therefore non-trivial and requires mapping of the energetics of the complete alternating-access cycle. Furthermore, sequence variations also result in more complex ion dependencies. For example, in brain cells NCKX catalyzes the co-transport of K^+^ and Ca^2+^ in exchange for Na^+^. This K^+^ dependence has been attributed to a single aspartate residue within the second α-repeats^34^. In mitochondria, the Na^+^/Ca^2+^ exchanger NCLX can also transport Li^+^ in exchange of Ca^2+^, and this transport mode is dependent on a single amino acid substitution in the second repeat^35^. Our results suggest that H256, conserved among many CAX proteins (and also in the second repeat) but replaced by leucine/threonine in Na^+^/Ca^2+^ exchangers (Supplementary Fig. 9), is the essential residue that enables Ca^2+^-P_i_ co-transport in YfkE. This histidine is not present in some prokaryotic CAXs, e.g., in ChaA, a CAX homolog from *E. coli*, a glycine residue is found at the equivalent position (Supplementary Fig. 9). ChaA, however, is distinct from YfkE in that it mediates H^+^-coupled K^+^ or Na^+^ efflux^36,37^. Thus, it seems plausible that Ca^2+^/P_i_ co-transport activity may be present in other CAX homologs. At any rate, our findings provide additional evidence that CaCA proteins alter their substrate selectivity by fine-tuning the α-repeat motifs used for Ca^2+^ recognition, resulting in different energizing or regulatory mechanisms in different cell types.

## Methods

### Preparation of inside-out vesicles

Inside-out vesicles (ISOs) were prepared by a low-pressure homogenization method. Briefly, *E. coli* BL21(DE3) cells harboring a pET28a-YfkE vector were grown in Luria broth medium at 37 °C to A_600_ of 0.4. Mutations were generated using a standard site-directed mutagenesis approach and confirmed by sequencing. Protein expression was induced by adding 0.2 mM isopropyl β-d-1-thiogalactopyranoside (IPTG) for 2 h at 25 °C. Cells were washed with buffer containing 10 mM Tris-HCl pH 7.3, 140 mM KCl, 0.5 mM DTT, 250 mM sucrose. Cell rupture was processed by a single passage through a C3 homogenizer (Avestin) with low pressure (4000 p.s.i.). After removing cell debris by centrifugation, supernatants were centrifuged using a Ti45 rotor at 40,000 rpm for 1 h to pellet membrane vesicles. Vesicles were homogenized in the same buffer and then quickly frozen in liquid nitrogen before use.

### Radioactive transport assays

Both Ca^2+^ and P_i_ transport activities of YfkE were measured using inside-out vesicles. Briefly, membrane vesicles were diluted in a buffer (pH 8.0) containing the same components. Before assays, an outward H^+^ gradient was established by adding 5 mM NADH to vesicles for 10 min. Although it is possible that a small number of right-side-out vesicles are present in our samples, the electron transport chain in these vesicles cannot be energized as NADH is membrane impermeable. Therefore, the only activity measured is that of the inside-out vesicles. For measuring Ca^2+^ transport activity, 5 mM cold potassium phosphate was also added to the vesicles. To measure phosphate transport activity, 0.5 mM cold CaCl_2_ was added instead. Transport assays were triggered by adding ^45^Ca^2+^ or ^32^P-P_i_ (Perkin Elmer) as indicated in the reactions at room temperature. Reactions were terminated by filtration through a nitrocellulose membrane (0.22 µm) on a Millipore filtration manifold and then washed immediately with buffer. The filters were air-dried and counted in a liquid scintillation counter to determine transport activity. Kinetic analysis was performed by data fitting into the Michaelis–Menten single exponential model using the software *Graphpad Prism* 9. For vesicle assays, membrane vesicles prepared with BL21(DE3) cells harboring empty vector were used as control. For kinetic assays of mutants, the concentration of vesicles was adjusted individually to obtain useful data for model fitting.

### Protein expression and purification

The proteins of YfkE WT and variants were expressed and purified using the same approach. Briefly, protein expression was carried out in *E. coli* C41(DE3) strain in auto-induction medium^38^ at 25 °C overnight. Cells were suspended in a buffer containing 20 mM Na phosphate, pH 7.4, 500 mM NaCl, and 20 mM imidazole and then ruptured by three passages through a C3 homogenizer (Avestin) at 15,000 p.s.i. Membrane fractions were collected as described above and suspended in lysis buffer. Membrane fractions were solubilized by adding 1% (w/v) n-dodecyl-β-maltoside (DDM) at 4 °C for 1 h and incubated with Ni-NTA resin (GE Healthcare). Resins were washed with buffer supplemented with 60 mM imidazole and 0.05% DDM and then eluted with buffer supplemented with 400 mM imidazole and 0.05% DDM. The eluted protein was further purified using a Superdex-200 10/300 GL column (GE Healthcare) equilibrated in a buffer containing 20 mM HEPES (pH 7.4), 500 mM NaCl and 0.05% DDM.

### Transport assays in reconstituted proteoliposomes

Proteoliposomes were prepared using *E. coli* total lipid (Avanti polar lipids Inc.) as follows: Lipids solubilized in chloroform were first dried under inert gas to form a thin lipid film in a glass tube and further kept under vacuum overnight to remove residual chloroform. The lipid film was then resuspended in 20 mM HEPES (pH 7.4), 100 mM NaCl and then sonicated on ice until the suspension became clear. After two freeze–thawing cycles, generated large multilamellar vesicles were passed through a 400 nm polycarbonate filter for 11 times to form unilamellar liposomes using a mini-extruder (Avanti Polar Lipids Inc). Prior to reconstitution, liposomes were first destabilized by adding 5 µl 10% Triton X-100 (wt/vol) per 1 mg lipids at room temperature for 30 min. The YfkE protein was mixed with detergent-destabilized liposomes in a 1:10 (*w*:*w*) ratio on ice for 20 min. Detergent was removed by adding bio-beads to the mixture (80 mg beads for 1 ml sample) to form proteoliposomes overnight at 4 °C with gentle agitation. Next day, fresh bio-beads were added for another 3 h to finalize the reconstitution.

To measure P_i_ transport activity, 200 µl 4 mg/ml YfkE-reconstituted proteoliposomes (pH = 7.4) were diluted into 400 µl buffer containing 20 mM HEPES (pH = 8.0), 100 mM NaCl, 5 mM ^32^P-phosphate (Perkin Elmer), and 0.5 mM CaCl_2_ (or 2 mM EDTA as control) at room temperature. At indicated times, reaction was terminated by filtration through a nitrocellulose membrane (0.22 µm) on a Millipore filtration manifold and then washed immediately with buffer. The filters were then air-dried and counted in a liquid scintillation counter to determine transport activity.

### Radiolabeled Pi binding assay

Prior to assays, 10 µg purified His-tag YfkE proteins were mixed with 100 µl Ni-NTA beads. 40 mM ^32^P_i_ was added to the beads in the presence of 0.5 mM Ca^2+^ for 10 min. The beads were then washed extensively using a protein buffer without P_i_. The proteins were eluted using 250 mM imidazole and the radioactivity was measured using a scintillation counter.

### Luminescence resonant energy transfer

Protein conformational changes in the native membrane environment were detected using LRET in ISOs. To ensure specific labeling, two endogenous cysteine residues (C34 and C293) were substituted with valine using site-directed mutagenesis to generate a cysteine-free YfkE construct. A pair of cysteine residues were then introduced for fluorophore labeling: one cysteine residue C3 was inserted at the N-terminus and another cysteine residue was placed at the position of 56 or 202 based on the YfkE structure. An amino-terminal flanking sequence immediately after C3 includes a His6 tag to increase the distance between the donor and receptor for optimal LRET signals and a thrombin cleavage site for background normalization.

ISO was prepared using the approach described above. Vesicle labeling was carried out using thiol-reactive chemistry for 1 h at room temperature in the dark using 200 nM maleimide derivative of donor and acceptor fluorophores. The fluorophores used are terbium chelate (Invitrogen) and Atto-465 (Sigma). After labeling, vesicles were dialyzed twice against a buffer containing 10 mM HEPES pH 7.3, 140 mM KCl, and 250 mM Sucrose for 2 h at 4 °C to remove excess probes. Before fluorescence scanning, 0.5 mM CaCl_2_ and/or 5 mM potassium phosphate (pH 7.3) were mixed with vesicles.

Fluorescence measurements were performed using a cuvette-based fluorescence lifetime spectrometer QuantaMaster model QM3-SS (Photon Technology International). Each experiment is reported as an average of three measurements for a given state, with each measurement having an average of 99 pulses from the flash lamp. Data were collected using Fluorescan software (Photon Technology International) and analyzed using *Origin* software (OriginLab Corp). The sensitized emission of the acceptor was detected before and after thrombin cleavage, a well-established technique used to subtract background fluorescence^39–41^. Specifically, after obtaining acceptor lifetime measurements, five units of bovine thrombin (Calbiochem) were added to the cuvette and allowed to cleave the N-terminal labeled fluorophore. Cleavage was completed 1–3 h following the addition of thrombin. The donor-acceptor labeled sample was excited at 337 nm, and the emission was detected at 508 nm. Distances were calculated using the Förster equation (Eq. (1)):

1 $${{{{{\rm{R}}}}}}={{{{{{\rm{R}}}}}}}_{0}{\left[\frac{{{{{{{\rm{\tau }}}}}}}_{{{{{{\rm{DA}}}}}}}}{{{{{{{\rm{\tau }}}}}}}_{{{{{{\rm{D}}}}}}}-{{{{{{\rm{\tau }}}}}}}_{{{{{{\rm{DA}}}}}}}}\right]}^{1/6}$$

### Isothermal titration calorimetry

Ca^2+^ and P_i_ binding affinity was determined using isothermal titration calorimetry (ITC). Proteins were decalcified by adding 10 mM EGTA and then separated using size-exclusion chromatography. The Ca^2+^ or P_i_ solution was prepared in the same buffer. ITC assays were performed by titrating Ca^2+^ into a solution of 5 or 10 μM YfkE protein on a VP-ITC device (Microcal LLC). All assays used the same ITC program: The system was thermally equilibrated at 25 °C; after an initial delay of 120 s, serial injections (10 μl each) were done with a spacing time of 240 s at 307 rpm stirring speed. Each measurement was corrected with a background titration in which the ligand was titrated into a buffer solution. Data fitting was carried out using *Origin* software (Microcal LLC).

### Molecular dynamics simulations and free-energy calculations

The simulations were based on the crystal structure of inward-facing YfkE (PDB 4KJR)^12^. All simulations were conducted with NAMD 2.9^42^ using the CHARMM36 force field for protein and lipids^43,44^. All simulations were carried out at constant temperature (298 K) and semi-isotropic pressure (1 atm), using periodic boundary conditions and an integration time step of 2 fs. Long-range electrostatic interactions were calculated using PME, with a real-space cut-off of 12 Å. Van der Waals interactions were computed with a Lennard-Jones potential, cut-off at 12 Å with a smooth switching function taking effect at 10 Å. The specific protein construct studied is a single protomer including residues 1–177 (TM-TM5) and residues 200-351 (TM6-TM10); the seemingly unstructured cytoplasmic loop connecting TM5 and TM6 was truncated. The protein construct was embedded in a pre-equilibrated hydrated palmitoyl-oleoyl-phosphatidyl-choline (POPC) lipid bilayer using GRIFFIN^45^ and enclosed in an orthorhombic box of ~88 × 88 × 95 Å in size. The resulting simulation system contains approximately 76,700 atoms, including 207 lipid molecules and a 100 mM KCl buffer (Supplementary Fig. 6). The simulation system was equilibrated following a staged protocol comprising a series of MD trajectories wherein positional and conformational restraints acting on the protein structure are gradually weakened over 100 ns. To evaluate the protonation state of a given sidechain in the protein against a reference of known pK_a_, an amino acid of the same type was included in the simulation system free in solution. This amino acid was neutralized at the N-terminus with an acetyl modification and at the C-terminus with a secondary-amide, so as to isolate the protonation energetics of the sidechain. Throughout the simulations, the free amino acid was maintained in solution and kept away from all other solutes (protein, membrane, ions) through a set of repulsive potentials acting at a certain threshold distance. Dual topologies (protonated and deprotonated) were introduced for both the protein sidechain and its equivalent in solution. The Free-Energy Perturbation (FEP) algorithm was then used to alter the protonation states of both sidechains, concurrently but in opposite directions; thus, the resulting free-energy value reflects the increase or decrease in the protonation propensity of the protein sidechain with respect to what is intrinsic for that sidechain type in solution (Supplementary Fig. 6). This transformation was carried out in both directions, i.e., protonation of the protein sidechain and deprotonation of the free amino acid, and vice versa. The ΔΔ*G* values shown in Table 2 reflect the mean value of these two calculations; the half-difference is provided as an error bar. Each calculation consisted in a series of consecutive MD simulations wherein a parameter l changes from 0 to 1 (or vice versa) as one topology is gradually replaced by the other, while the corresponding changes in potential energy are annotated for each simulation snapshot. Each l transformation was discretized in 40 steps, each of which consisted of a simulation of 1 ns, for sidechains in protein interior, or 240 ps, for the more mobile surface sidechains. These 40 simulations were carried out sequentially; after each incremental change in l, a trajectory fragment (200 or 40 ps, respectively) was considered as equilibration time and omitted from the free-energy calculation.

### Survey of the protein data bank

We analyzed protein X-ray structures that fulfill the following criteria: (i) the resolution is 3.0 Å or better; (ii) the sequence identity with a previously selected structure is lower than 70%; (iii) the structures have one calcium and one phosphate ion bound, in a common binding site (i.e., the minimum distance between the two ions is 3.2 Å or shorter); and (iv) the binding site is flanked by two acidic and one histidine side-chain, like in YfkE. This survey resulted in one structure, namely that of the mammalian serum paraoxonase 1 (PON1), a calcium-dependent hydrolase (PDB entry 3SRE)^46^.

### Structure modeling

The molecular model of YfkE bound to Ca^2+^ and phosphate was generated with MODELLER v9.13^47^. The procedure followed was akin to that used in homology modeling, except that here the target was the unknown structure of the Ca^2+^/phosphate-bound state, and the template was the known structure (PDB 4KJR)^12^. Only the structure of the four TM helices flanking the binding site (the so-called α-repeats, i.e., residues 59–110 and 239–296) was remodeled. To specifically model the geometry of the binding site, with Ca^2+^ and phosphate bound, we added a set of geometric restraints inferred from the abovementioned structures of PON1 and NCX_Mj^16,30^, and Ca^2+^/phosphate transport studies of wild-type and mutated forms of YfkE. Specifically, the latter have shown that the following mutations cause significant changes in measured *K*_M_ and/or *V*_max_ values, without abrogating transport: G68A, N69A, N99A, N252A, H256A, and Q281A. Thus, restraints were applied to the following distances between Ca^2+^ and the protein, using a harmonic potential acting at distances equal to or greater than 2.6 Å (and a ‘deviation’ of 0.1 Å): (1) to each of the four oxygen atoms from the carboxylate groups of E72 and E255; (2) to the nearest of the oxygen atoms of phosphate (modeled as H_2_PO_4_^−^); (3) to the carbonyl of G68; and (4) to the carbonyl oxygen of the N69 sidechain. Taken together, these four restraints imply a Ca^2+^ coordination number of 7. Analogous restraints were applied to the distances between the phosphorus atom and (5) Nδ of H256 at 3.9 Å, (6) Cγ of N252 (4.5 Å), and (7) Cγ of S278 (4.5 Å); and to the distance between (8) Oε of Q281 and Nδ of N69 (3.2 Å).; and (9) that between Cγ of N252 and Cγ of N99 (4.2 Å). Finally, to impose double-bi-dentate interactions between Ca^2+^ and E72 and E255, a restraint was also applied to (10) the dihedral angles formed by Ca^2+^ and atoms Cδ, Oε1, and Oε2 of E72 and E255 (0°, with ~20° deviation). We generated an ensemble of 2000 models in total and analyzed this ensemble through clustering based on pairwise RMSD; this RMSD calculation included all non-hydrogen atoms of residues N69, E72, N99, N252, E255, H256, S278, and Q281, plus the Ca^2+^ and phosphate ions, and clusters were defined by an RMSD threshold value of 0.6 Å. 15 clusters comprising more than 10 models resulted from this analysis, which add up to 92% of the models.

### Statistics and reproducibility

All experiments were performed at least three times (*n* = 3) to ensure data reproducibility. All data points and error bars (standard deviations) are provided in each figure and Supplementary Data if applicable.

### Reporting summary

Further information on research design is available in the Nature Portfolio Reporting Summary linked to this article.

## Supplementary information

Supplementary Material

Description of Additional Supplementary Data

Supplementary Data 1

Reporting Summary

## Data Availability

All data generated or analyzed during this study are included in this published article (and its Supplementary Information files). Source data for figures can be found in Supplementary Data 1.

## Code availability

Files for MD simulation are available at https://github.com/Faraldo-Gomez-Lab-at-NIH/Download.
