# Supplementary material for: Ca2+ efflux facilitated by co-transport of inorganic phosphate anion in the H+/Ca2+ antiporter YfkE
Source: Commun Biol. 2023 May 29;6:573. doi: 10.1038/s42003-023-04944-6 (PMC10227063; doi:10.1038/s42003-023-04944-6)
Supplement: Supplementary file 1 — Supplementary Material [file 42003_2023_4944_MOESM1_ESM.pdf]

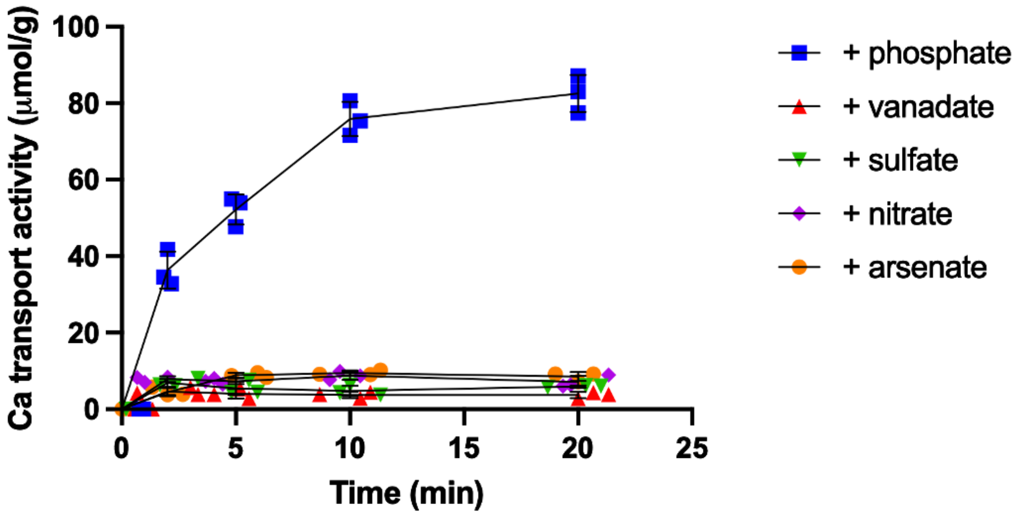

Supplementary Fig. 1: Specificity of inorganic phosphate ion in facilitating Ca^{2+} efflux of YfkE. Ca^{2+} transport assays were measured using inside-out vesicles. Before assays, vesicles were incubated with 5mM potassium phosphate, sodium sulfate, sodium nitrate, sodium arsenate, or sodium vanadate for 10 min before 0.1mM $^{45}Ca^{2+}$ was added to trigger the reactions. The reactions were stopped at indicated times to measure radioactivity by scintillation counting. Error bars represent standard deviation (n=3).

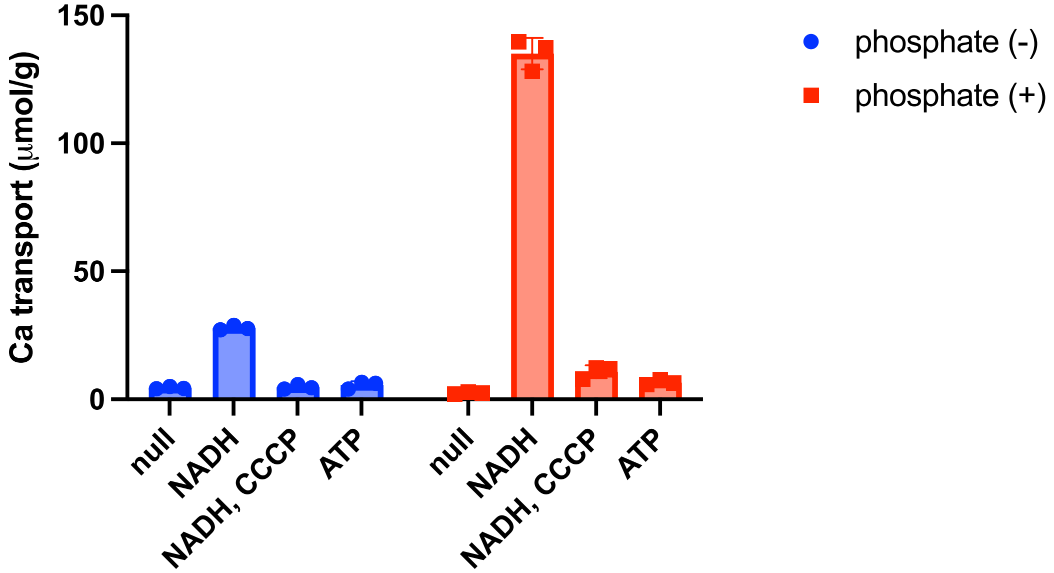

Supplementary Fig. 2: H^+ coupling of Ca^{2+} transport of YfkE in both phosphate-independent and phosphate-dependent transport modes. Ca^{2+} transport activity was performed using inside-out vesicles. Before assays, NADH, H^+ ionophore carbonyl cyanide m-chlorophenyl hydrazone (CCCP), or ATP was added to vesicles in the presence or absence of 5mM potassium phosphate for 10min at room temperature. The reactions were triggered by adding 0.5mM $^{45}CaCl_2$ for 20min. The radioactivity was measured by scintillation counting. Error bars represent standard deviation (n=3).

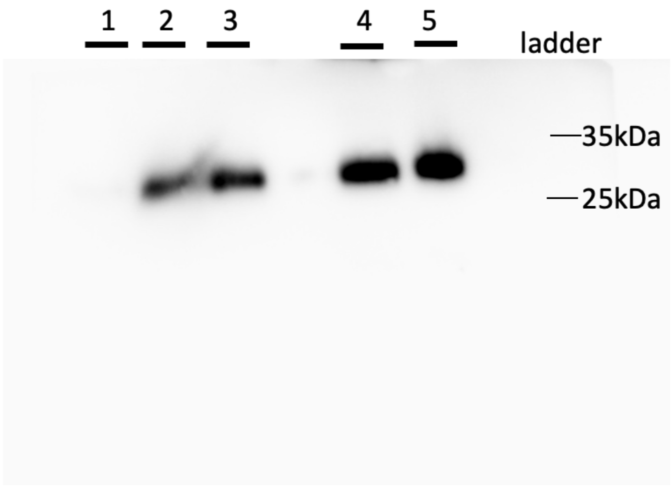

Supplementary Fig. 3: Raw image of the immunoblot of YfkE-reconstituted proteoliposomes. 1&5) the YfkE protein; 3) YfkE vesicles; 2&4) YfkE-reconstituted proteoliposomes. Samples treated with thrombin (1&4) or without treatment (2&5). Samples were loaded on a 15% SDS-PAGE gel. The blot was then developed using an anti-His tag antibody and an anti-mouse antibody-HRP conjugate. The image was captured using a Bio-Rad imaging system. The samples #4 and #5 are inverted in Fig. 2c for clarity.

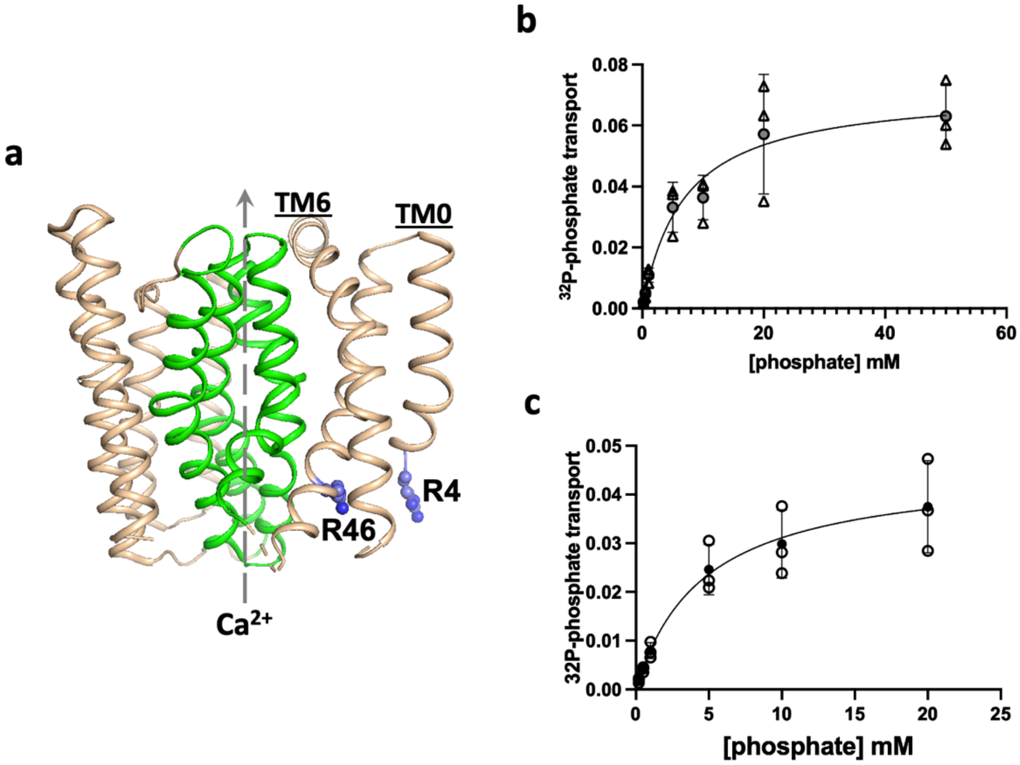

Supplementary Fig. 4: Mutational analysis of positively charged arginine residues on the intracellular surface of YfkE. a Overview of the YfkE structure showing R4 from TM0 and R46 from TM1 (*blue stick-balls*) on the intracellular surface. The only polar Ca^{2+} translocation pathway formed by four helices in the middle of the protein was colored in *green*, others in *grey*. (**b** and **c**) ^{32}P-phosphate transport kinetic assays of YfkE mutants R4A (**b**) and R46A (**c**) were measured in the presence of 0.5mM $CaCl_2$. Data fitting into Michaelis-Menten kinetics model using GraphPad Prism 9. Error bars represent standard deviations (n=3).

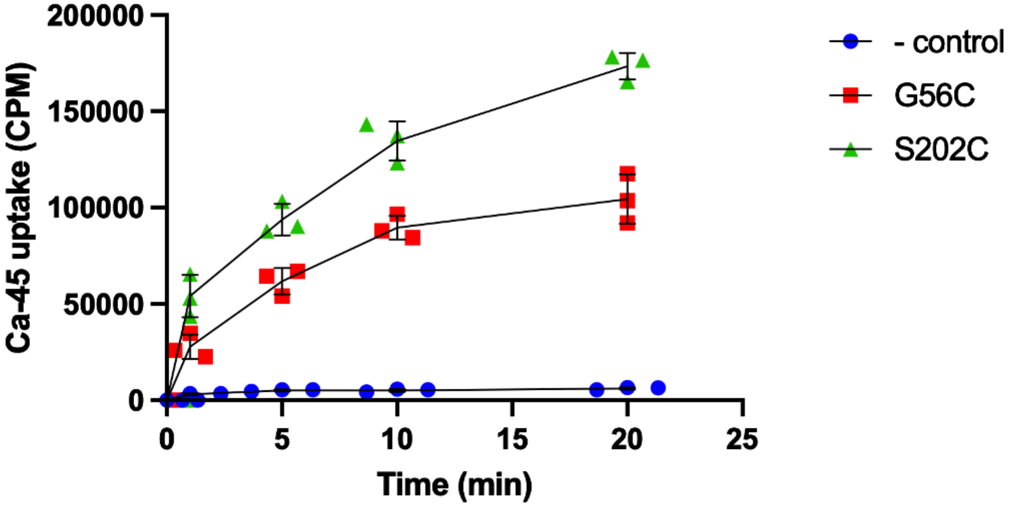

Supplementary Fig. 5: Ca^{2+} transport assays of two cysteine mutants of YfkE used in LRET experiments measured using inside-out vesicles. The assays show that radiolabeled ^{45}Ca (CPM) was imported into vesicles by mutants G56C and S202C in the presence of 5mM P_i in contrast to control vesicles. Error bars represent standard deviations (*n*=3).

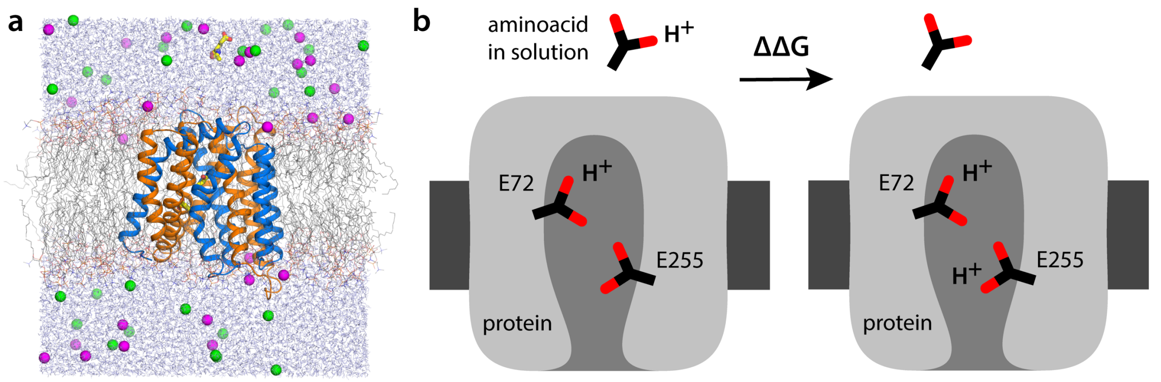

Supplementary Fig. 6: Protonation free-energy calculations based on MD simulations. **a** Molecular simulation system, comprising a single protomer of inward-facing YfkE (cartoons, with TM1-TM5 in orange and TM6-TM10 in blue), embedded in a POPC bilayer and a 100 mM KCl buffer (green/magenta). Residues E55 and E255 within the protein interior are highlighted, as is a glutamate amino acid-free in solution, which we use as a reference of known pK_a (yellow). (**b**) Schematic representation of the methodology followed to evaluate the protonation states of selected residues within the protein. In the example depicted, simulations are used to evaluate the free-energy gain or loss upon protonation of E255, relative to the opposite reaction for the glutamate sidechain in solution, in the context of protonated E72. A free-energy gain (negative DDG) would indicate that the protein environment, in the structure considered, increases the propensity for protonation of E255, relative to what would be expected for a sidechain of the same type in solution. Note that the free amino acid in solution is acetylated at the N-terminus and secondary-amidated at the C-terminus, to mimic the protein backbone (Methods); hence the calculated DDG value is defined in reference to the solution pK_a value of the sidechain. For example, a DD*G* value approximately equal to –1.4 kcal/mol corresponds to a pK_a shift of +1 pH unit. Analogous schemes were used to calculate all the values reported in Table 2, substituting glutamate with histidine as needed.

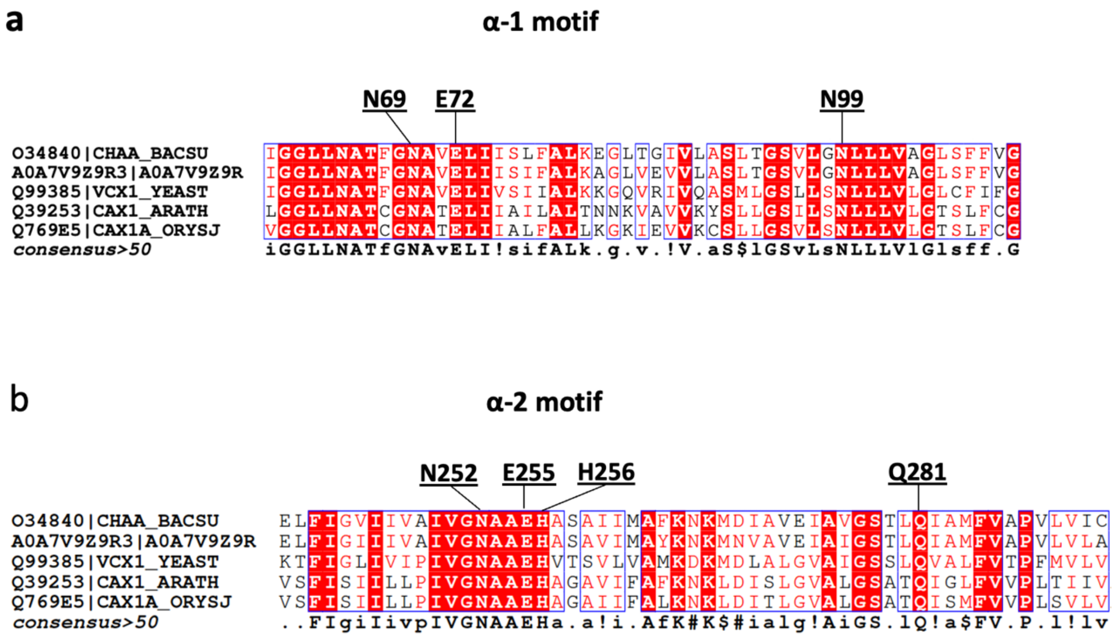

Supplementary Fig. 7: Sequence alignment of the two α-repeat sequences of the CAX family proteins showing conserved residues in the Ca^{2+}-P_i binding site. a α-1 motif; **b** α-2 motif. The protein sequences in the alignment are: YfkE from *Bacillus subtilis* (O34840 | CHAA_BACSU), CAX from *Anoxybacillus caldiproteolyticus* (A0A7V9Z9R3_9BACI), VCX1 from *Saccharomyces cerevisiae* (Q99385 | VCX1_YEAST), CAX1 from *Arabidopsis thaliana* (Q39253 | CAX1_ARATH), CAX1a from Rice *Oryza sativa subsp. japonica* (Q769E5 | CAX1A_ORYSJ), The amino acid residues in the putative Ca-P_i binding site of YfkE are highlighted. The sequence alignment was generated using the program Multalin (1) and the figure was generated by the program ESPript (2)

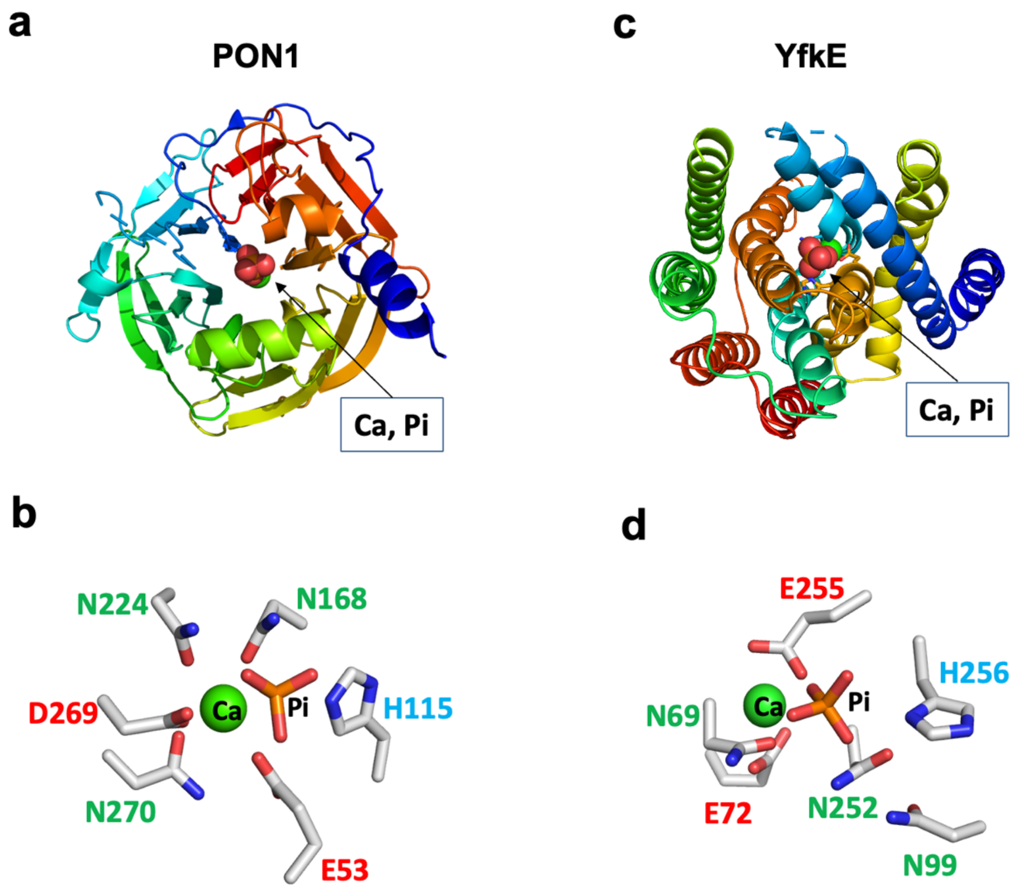

Supplementary Fig. 8: Comparison between the binding sites for Ca^{2+} and inorganic phosphate ion (Pi) in the crystal structure of the Ca^{2+}-dependent hydrolase PON1 and in the proposed model of inward-facing YfkE. a Overview of the PON1 structure (PDB entry 3SRE), showing a donut-shaped conformation with both Ca^{2+} and Pi (*green* or *red spheres*) bound adjacently in the central pore. **b** Zoom view of the binding site conformation of PON1 showing Ca^{2+} and P_i are coordinated concurrently by five residues including two carboxylate residues (E53 and D269), three asparagine residues (N168, N224, and N270), and one histidine residue (H115). **c** Overview of the putative structural model of YfkE showing Ca^{2+} and P_i (*green* or *red spheres*) in the central translocation pathway. **d** Zoom view of the putative Ca^{2+}- P_i binding site in the YfkE structural model showing a side-chain configuration similar to that of PON1, which is comprised of five amino acid residues including two carboxylate residues (E72 and E255), three asparagine residues (N69, N99, and N252), and one histidine residue (H256). These residues are labeled: carboxylate (*red*), asparagine (*green*), and histidine (*blue*).

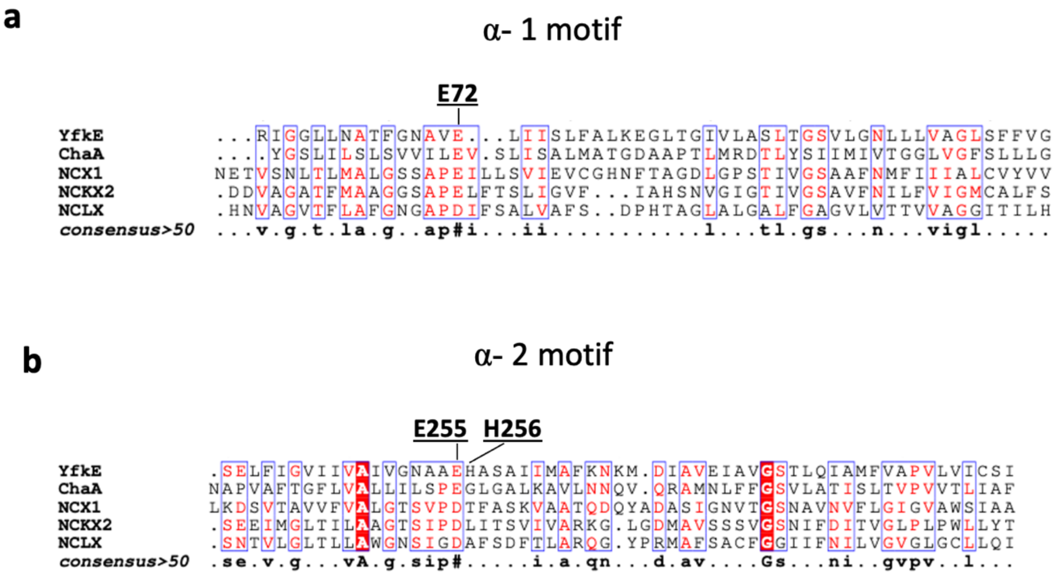

Supplementary Fig. 9: Sequence alignment of the two α-repeat motifs of Ca2+ cation antiporter protein superfamily members. **a** α-1 motif region; **b** α-2 motif region. The protein sequences in the alignment are: H^+/Ca^{2+} antiporter YfkE from *Bacillus subtilis* (O34840 | CHAA_BACSU), H^+/Na^+ or K^+ antiporter ChaA from *E. coli* (P21801_ECOLI), Na^+/Ca^{2+} exchanger NCX1 from human (P32418 | NAC1_HUMAN), Na^+, K^+/Ca^{2+} exchanger NCKX2 from human (Q9UI40 | NCKX2_HUMAN), mitochondrial sodium/calcium exchanger NCLX from human (Q6J4K2 | NCLX_HUMAN). The sequence alignment was generated using the program Multalin (1) and the figure was generated by the program ESPript (2). The conserved carboxylate residues E72 and E255, and H256 from YfkE are labeled.

References

1. Corpet, F. (1988) Multiple sequence alignment with hierarchical clustering. Nucleic Acids Res 16, 10881-10890
2. Robert, X., and Gouet, P. (2014) Deciphering key features in protein structures with the new ENDscript server. Nucleic Acids Research 42, W320-W324
