# Supplementary material for: Ca2+ efflux facilitated by co-transport of inorganic phosphate anion in the H+/Ca2+ antiporter YfkE
Source: Commun Biol. 2023 May 29;6:573. doi: 10.1038/s42003-023-04944-6 (PMC10227063; doi:10.1038/s42003-023-04944-6)
Supplement: Supplementary file 4 — Reporting Summary [file 42003_2023_4944_MOESM4_ESM.pdf]

nature research

Corresponding author(s): José D. Faraldo-Gómez and Lei Zheng

Last updated by author(s): May 2, 2023

Reporting Summary

Nature Research wishes to improve the reproducibility of the work that we publish. This form provides structure for consistency and transparency in reporting. For further information on Nature Research policies, see our Editorial Policies and the Editorial Policy Checklist.

Statistics

For all statistical analyses, confirm that the following items are present in the figure legend, table legend, main text, or Methods section.

n/a	Confirmed	
☐	☒	The exact sample size (n) for each experimental group/condition, given as a discrete number and unit of measurement
☐	☒	A statement on whether measurements were taken from distinct samples or whether the same sample was measured repeatedly
☒	☐	The statistical test(s) used AND whether they are one- or two-sided<br>*Only common tests should be described solely by name; describe more complex techniques in the Methods section.*
☒	☐	A description of all covariates tested
☒	☐	A description of any assumptions or corrections, such as tests of normality and adjustment for multiple comparisons
☒	☐	A full description of the statistical parameters including central tendency (e.g. means) or other basic estimates (e.g. regression coefficient) AND variation (e.g. standard deviation) or associated estimates of uncertainty (e.g. confidence intervals)
☒	☐	For null hypothesis testing, the test statistic (e.g. F, t, r) with confidence intervals, effect sizes, degrees of freedom and P value noted<br>*Give P values as exact values whenever suitable.*
☒	☐	For Bayesian analysis, information on the choice of priors and Markov chain Monte Carlo settings
☒	☐	For hierarchical and complex designs, identification of the appropriate level for tests and full reporting of outcomes
☒	☐	Estimates of effect sizes (e.g. Cohen's d, Pearson's r), indicating how they were calculated

Our web collection on statistics for biologists contains articles on many of the points above.

Software and code

Policy information about availability of computer code

Data collection	Origin (MicroCAL) for ITC, Fluorescan software (Photon Technology International) for LRET experiments
Data analysis	Prism Graphpad 9, Origin (OriginLab Corp), NAMD 2.9, and MODELLER v9.13

For manuscripts utilizing custom algorithms or software that are central to the research but not yet described in published literature, software must be made available to editors and reviewers. We strongly encourage code deposition in a community repository (e.g. GitHub). See the Nature Research guidelines for submitting code & software for further information.

Data

Policy information about availability of data

All manuscripts must include a data availability statement. This statement should provide the following information, where applicable:

- Accession codes, unique identifiers, or web links for publicly available datasets
- A list of figures that have associated raw data
- A description of any restrictions on data availability

All data in the manuscript are included in the main text and supplementary information.

Field-specific reporting

Life sciences study design

All studies must disclose on these points even when the disclosure is negative.

Sample size	The sample size was determined based on the previous publications by our lab. No software was used to determine sample size.
Data exclusions	No data exclusions in the study.
Replication	All assays were performed for at least three times as described in the manuscript. All data points are included in figures.
Randomization	This work only contains in vitro protein biochemical study. Randomization is not applicable.
Blinding	This work only contains in vitro protein biochemical study. Experimental design is based on the crystal structure. Blinding is not applicable.

Reporting for specific materials, systems and methods

We require information from authors about some types of materials, experimental systems and methods used in many studies. Here, indicate whether each material, system or method listed is relevant to your study. If you are not sure if a list item applies to your research, read the appropriate section before selecting a response.

Materials & experimental systems

n/a	Involved in the study	
☐	☒	Antibodies
☒	☐	Eukaryotic cell lines
☒	☐	Palaeontology and archaeology
☒	☐	Animals and other organisms
☒	☐	Human research participants
☒	☐	Clinical data
☒	☐	Dual use research of concern

Methods

n/a	Involved in the study	
☒	☐	ChIP-seq
☒	☐	Flow cytometry
☒	☐	MRI-based neuroimaging

Antibodies

Antibodies used	anti-His tag antibody purchased from Lifetein Inc. https://www.lifetein.com/index.html
Validation	This antibody is to used to detect a His tag protein. Validation from the manufacturer and cited publications can be found in the product website: https://www.lifetein.com/peptide-product/anti-his-monoclonal-antibody-p-2.html
